# Genome-wide association study of angioedema induced by angiotensin-converting enzyme inhibitor and angiotensin receptor blocker treatment

**DOI:** 10.1038/s41397-020-0165-2

**Published:** 2020-02-21

**Authors:** Eva Rye Rasmussen, Pär Hallberg, Ekaterina V. Baranova, Niclas Eriksson, Malgorzata Karawajczyk, Caroline Johansson, Marco Cavalli, Cyrielle Maroteau, Abirami Veluchamy, Gunilla Islander, Svante Hugosson, Ingrid Terreehorst, Folkert W. Asselbergs, Pia Norling, Hans-Erik Johansson, Hugo Kohnke, Ann-Christine Syvänen, Moneeza K. Siddiqui, Chim C. Lang, Patrik K. E. Magnusson, Qun-Ying Yue, Claes Wadelius, Christian von Buchwald, Anette Bygum, Ana Alfirevic, Anke H. Maitland-van der Zee, Colin N. A. Palmer, Mia Wadelius

**Affiliations:** 1 Department of Otorhinolaryngology, Head & Neck Surgery and Audiology, Rigshospitalet, University of Copenhagen, Copenhagen, Denmark; 2 OPEN Patient data Explorative Network, Odense University Hospital, Odense, Denmark; 3 Department of Medical Sciences, Clinical Pharmacology and Science for Life Laboratory, Uppsala University, Uppsala, Sweden; 4 Department of Pharmaceutical Sciences, Division of Pharmacoepidemiology and Clinical Pharmacology, Utrecht University, Utrecht, The Netherlands; 5 Uppsala Clinical Research Center, Uppsala, Sweden; 6 Department of Medical Sciences, Clinical Chemistry, Uppsala University, Uppsala, Sweden; 7 Q-Linea AB, Uppsala, Sweden; 8 Department of Immunology, Genetics and Pathology, Medical Genetics and Genomics and Science for Life Laboratory, Uppsala University, Uppsala, Sweden; 9 Division of Population Health and Genomics, University of Dundee, Ninewells Hospital and Medical school, Dundee, UK; 10 Department of Intensive and Perioperative Care, Skåne University Hospital, Lund, Sweden; 11 Department of Otorhinolaryngology, Örebro University Hospital and Örebro University, Örebro, Sweden; 12 Department of Ear, Nose and Throat diseases, Amsterdam University Medical Center, Amsterdam, The Netherlands; 13 Department of Cardiology, Division Heart and Lungs, University Medical Center Utrecht, Utrecht, The Netherlands; 14 Institute of Cardiovascular Science, Faculty of Population Health Sciences, University College London, London, UK; 15 Health Data Research UK and Institute of Health Informatics, University College London, London, UK; 16 Sickla Health Centre, Nacka, Sweden; 17 Department of Public Health and Caring Sciences, Clinical Nutrition and Metabolism, Uppsala University, Uppsala, Sweden; 18 Department of Medical Sciences, Molecular Medicine and Science for Life Laboratory, Uppsala University, Uppsala, Sweden; 19 Division of Molecular & Clinical Medicine, Ninewells Hospital and Medical School, University of Dundee, Dundee, UK; 20 Swedish Twin Registry, Department of Medical Epidemiology and Biostatistics, Karolinska Institutet, Stockholm, Sweden; 21 Uppsala Monitoring Centre, WHO Collaborating Centre, Uppsala, Sweden; 22 Department of Dermatology and Allergy Centre, Odense University Hospital, Odense, Denmark; 23 Clinical Institute, University of Southern Denmark, Odense, Denmark; 24 Department of Molecular & Clinical Pharmacology, University of Liverpool, Liverpool, UK; 25 Department of Respiratory Diseases, Amsterdam University Medical Center, University of Amsterdam, Amsterdam, The Netherlands

**Keywords:** Risk factors, Genetic association study

## Abstract

Angioedema in the mouth or upper airways is a feared adverse reaction to angiotensin-converting enzyme inhibitor (ACEi) and angiotensin receptor blocker (ARB) treatment, which is used for hypertension, heart failure and diabetes complications. This candidate gene and genome-wide association study aimed to identify genetic variants predisposing to angioedema induced by these drugs. The discovery cohort consisted of 173 cases and 4890 controls recruited in Sweden. In the candidate gene analysis, *ETV6, BDKRB2, MME*, and *PRKCQ* were nominally associated with angioedema (*p* < 0.05), but did not pass Bonferroni correction for multiple testing (*p* < 2.89 × 10^−5^). In the genome-wide analysis, intronic variants in the calcium-activated potassium channel subunit alpha-1 (*KCNMA1*) gene on chromosome 10 were significantly associated with angioedema (*p* < 5 × 10^−8^). Whilst the top *KCNMA1* hit was not significant in the replication cohort (413 cases and 599 ACEi-exposed controls from the US and Northern Europe), a meta-analysis of the replication and discovery cohorts (in total 586 cases and 1944 ACEi-exposed controls) revealed that each variant allele increased the odds of experiencing angioedema 1.62 times (95% confidence interval 1.05–2.50, *p* = 0.030). Associated *KCNMA1* variants are not known to be functional, but are in linkage disequilibrium with variants in transcription factor binding sites active in relevant tissues. In summary, our data suggest that common variation in *KCNMA1* is associated with risk of angioedema induced by ACEi or ARB treatment. Future whole exome or genome sequencing studies will show whether rare variants in *KCNMA1* or other genes contribute to the risk of ACEi- and ARB-induced angioedema.

## Introduction

Angiotensin-converting enzyme inhibitor (ACEi) and angiotensin receptor blocker (ARB) containing medications are often used for hypertension, heart failure and diabetes complications, and currently 14% of the population in Sweden is treated with these drugs [1]. Although being mostly effective and safe, around 0.2–0.7% of patients starting an ACEi, and 0.1–0.2% starting an ARB experience the adverse drug reaction (ADR) angioedema [2–6]. Compared with other populations, the risk is higher in African Americans, suggesting a genetic effect modification [6–8]. The reaction is not dose-related and displays large discrepancies in time to onset, ranging from hours to years [9, 10].

ACEi- and ARB-induced angioedema is characterised by swelling of the reticular dermis and subcutaneous skin layers or mucosa [9, 11, 12]. It is usually localised to the head and neck region, and may become life threatening when breathing is impaired due to swelling in the oral cavity, tongue, pharynx or larynx [12]. In rare instances the intestines or genitals may be involved [13]. The pathophysiological mechanism is believed to involve the accumulation of the vasoactive peptide bradykinin [11, 12]. ACEis directly inhibit the inactivation of bradykinin while blocking the conversion of angiotensin I to angiotensin II [14]. ARBs have no direct effect on bradykinin [15], but may increase bradykinin levels through indirect inhibition of ACE and metallo-endopeptidase [16]. Drugs conventionally used in immune-mediated angioedema such as antihistamines, glucocorticoids and adrenaline have limited effects on ACEi- and ARB-induced angioedema [12, 17]. Alternative treatments are the bradykinin receptor antagonist icatibant, complement C1-esterase inhibitors, ecallantide, and fresh frozen plasma, but definite proof of their benefit is still lacking [12, 18, 19].

Two recent reviews describe nine studies with possible genetic associations to ACEi-induced angioedema [20, 21]. The majority are case–control studies that focus on candidate genes in bradykinin pathways. Three studies found significant associations with variation in the bradykinin-degrading enzyme aminopeptidase P gene *XPNPEP2* [22–24], and one found an association with a variant of the type 2 bradykinin receptor gene (*BDKRB2)* [25]. Mutations in serpin super family and clotting factor genes causing the rare condition hereditary angioedema are also of interest [26]. It is recommended that these patients avoid ACEis [27], but a mutation in the Serpin Family E Member 1 gene (*SERPINE1*) has been described in at least one patient with apparent ACEi-induced angioedema [28], and a mutation in the Coagulation Factor XII gene (*F12*) in another [29]. None of the mentioned candidate genes was significantly associated in the only published genome-wide association study (GWAS) on ACEi-induced angioedema [30]. The main findings were a non-significant association between risk of angioedema and the ETS Variant Transcription Factor 6 gene (*ETV6*) in African Americans, while a variant of the Protein Kinase C Theta gene (*PRKCQ*) tended to be protective in European Americans [30]. Furthermore, in a candidate gene analysis of the same study, a nominal association with the bradykinin degrading enzyme Neprilysin gene (Membrane Metalloendopeptidase, *MME*) was detected in African Americans [30]. Neprilysin-inhibitors are a recent addition to the arsenal of agents used in cardiovascular disease, and are known to augment the risk of angioedema in ACEi-treated patients [31, 32].

The objective of this genome-wide association and candidate gene study was to investigate whether any genetic variants predispose to angioedema associated with ACEi or ARB treatment in a Northern European population, and if so to assess the functionality of detected associations.

## Subjects and methods

### Ethics statement

Research was carried out in accordance with the latest update of the Declaration of Helsinki. Written informed consent was obtained from all participants in the discovery and replication cohorts. The study protocols were approved by Ethics Committees or Institutional Review Boards in each country. Please see the Supplementary Methods for details.

### Discovery cohort description

Angioedema cases associated with ACEi or ARB treatment reported to the Swedish national ADR registry at the Medical Products Agency or referred from collaborating clinicians were recruited according to SWEDEGENE’s standardised procedure (www.swedegene.se). Please see the Supplementary Methods for details. Inclusion and exclusion was according to published phenotype criteria [11]. All cases were reviewed and adjudicated by a clinical expert in allergology. Patients provided an EDTA blood sample that was stored at −70 °C until DNA extraction. Unrelated population controls were obtained from the Swedish Twin registry. The subgroup treated controls had collected at least two prescriptions of an ACEi at a Swedish pharmacy according to the Swedish Prescribed Drug Register [1, 33]. Indications for treatment were searched for in the Swedish National Patient Register, but were only available for 71% of the controls since the register has a low sensitivity for primary care diagnosis [34]. Controls who had been given the diagnoses angioedema or larynx-oedema were excluded [35].

### Discovery genotyping, imputation and data analysis

Cases were genotyped on the Illumina HumanOmni2.5 BeadChip array and controls had previously been genotyped on the Illumina HumanOmniExpress 700 K BeadChip (Illumina, CA, USA). Quality control and data management was done with PLINK v1.9 [36]. Sex chromosomes were filtered out at an early stage. The resulting merged data set included 622992 single nucleotide polymorphisms (SNPs). Imputation of genotypes was performed with PhaseIT [37] and Impute v2 using 1000 genomes as reference [38]. Post-imputation, variants with MAF below 1% or a missing rate above 2%, and individuals with more than 2% missing markers were filtered out. SNPs deviating from the Hardy-Weinberg equilibrium (*p* < 10^−6^) among controls were removed. The final dataset contained 7585599 SNPs. The quantile–quantile (Q-Q) plot for analysis of cases vs all controls after imputation is shown in Supplementary Fig. S1. Principal component analysis was performed on non-imputed data in order to account for possible population stratification. Logistic regression was used to estimate univariate and multiple models. SNP effects were modelled as additive. The conventional genome-wide significance threshold *p* < 5 × 10^−8^ was used to correct for multiple testing [39]. PLINK v1.9 was used for genome-wide analyses, and R 3.2.2 (R Foundation for Statistical Computing, Vienna, Austria) for individual SNP analyses.

In addition, we performed candidate gene analyses for *BDKRB2, ETV6, F12, MME, PRKCQ*, and *SERPINE1* in the imputed data set [20, 21]. All variants within the chromosomal positions (Table 1) ±10,000 bases upstream and downstream were extracted. *XPNPEP2*, which is located on the X chromosome, could not be evaluated since sex chromosomes were filtered out pre-imputation. SNP effects were modelled as additive. The number of tested genetic variants was 1730, and the Bonferroni cut-off for correction for multiple testing was *p* < 2.89 × 10^−5^ (= 0.05/1730 SNPs). All 173 cases were tested vs 4890 unmatched controls, and vs the 1345 matched controls.

**Table 1:** Candidate genes for angiotensin-converting enzyme inhibitor-induced angioedema tested in the study. Chromosomal position is according to the Genome Reference Consortium Human genome build 37.

Genes	Chromosome	Start position	End position
*BDKRB2*	14	96671016	96710666
*ETV6*	12	11802788	12048336
*F12*	5	176829141	176836577
*MME*	3	154741913	154901497
*PRKCQ*	10	6469105	6622263
*SERPINE1*	7	100770370	100782547

The genes encode Bradykinin Receptor B2 (*BDKRB2*), ETS Variant Transcription Factor 6 (*ETV6*), Coagulation Factor XII (*F12*), Neprilysin or Membrane Metalloendopeptidase (*MME*), Protein Kinase C Theta (*PRKCQ*), and Serpin Family E Member 1 (*SERPINE1*). All variants within the chromosomal positions plus 1000 bases upstream and 1000 bases downstream were extracted.

### Power calculation

Our sample size was powered to detect common genetic variants with effect sizes of clinical utility [40]. Power calculations were based on an ADR prevalence of 1%, and a minor allele frequency (MAF) of 10%, 173 cases, 4890 unmatched and 1345 matched controls, and an additive genetic model. Given a genome-wide significance level of 5 × 10^−8^, we had 80% power to detect an odds ratio (OR) of 3.0 when using unmatched controls, and 80% power to detect an OR of 3.1 when using matched controls (Supplementary Fig. S2a, b). Given a candidate gene significance level of 2.89 × 10^−5^, we had 80% power to detect an OR of 2.4 when using unmatched controls, and 80% power to detect an OR of 2.5 when using matched controls (Supplementary Fig. S3a, b).

### Replication cohort description

Data from the published ACEi-angiodema GWAS cohort from Vanderbilt and Marshfield, USA, was obtained through collaboration [30]. Cases were defined as patients who developed swelling of the lips, pharynx or face for the first time while taking an ACE inhibitor. Controls had been treated with an ACEi for at least 6 months without developing angioedema.

The Northern European cohort was recruited in four countries, and was part of the PREDICTION-ADR study. Please see the Supplementary Methods for details. In Sweden, cases were recruited according to SWEDEGENE’s standardised procedure. In Denmark, cases were referred by physicians at participating hospitals and a general practitioner. In the Netherlands, cases were referred by physicians at participating hospitals or identified by screening electronic medical records from all hospital admissions for angioedema. In the UK, cases were identified by referral from consultant immunologists, and by review of archived clinical letters. The expert who assessed the discovery cases also assessed all replication cases and used identical criteria [11]. Treated controls from Sweden were recruited by collaborating clinicians. Danish treated controls were selected from existing databases [41]. Dutch treated controls were participants of Utrecht Cardiovascular Pharmacogenomics (UCP) studies [42]. British treated controls were patients in the Genetics of Diabetes Audit and Research Tayside Study (GoDARTS) [43]. Cases and controls provided a blood or saliva sample (2 ml Oragene® OG-500 collection kit, DNA Genotek, Canada). Blood samples were kept at −70 °C and saliva samples at room temperature until DNA extraction according to standard procedures.

### Replication genotyping and meta-analysis

The American cohort was genotyped using a 610Quadv1.B Illumina Bead Chip. Genotyping of the Northern European replication cohort was performed on the Applied Biosystems 7500 Fast Real-Time PCR System (Thermo Fisher Scientific, Waltham, Massachusetts, USA) according to standard procedures. The TaqMan SNP Genotyping Assay kit for rs2253202 (C__15924034_20) was used.

Meta-analysis was performed using one genotyped SNP in the Northern European replication cohort, and summary data for this SNP in the published American GWAS [30] and the discovery GWAS. Logistic regression was used to evaluate the association between the SNP and angioedema. The SNP effect was modelled as additive. Meta-analysis was performed using a fixed-effect model in the metafor R-package [44]. The cut-off for a statistically significant association was set to *p* < 0.05.

### Functional analysis

Functional annotations were obtained by intersecting top GWAS SNPs and SNPs in high linkage disequilibrium (LD) with transcription factor binding sites reported by the ENCODE project, and with chromatin state models from the Roadmap Epigenomics project [45–47]. Chromatin state was based on deoxyribonuclease (DNAse) I hypersensitive clusters and histone modifications that indicate active regulatory regions (H3K4me3 and H3K27ac). We used annotations in skin, mucosa-derived tissues, oesophagus, aorta, smooth muscle and the umbilical vein endothelial cell line HUVEC. Motif analysis was based on a position weight matrix library and enhancer tissue-specific motif clustering [47–49].

## Results

### Discovery cohort characteristics

One hundred and seventy-three cases of angioedema associated with ACEi and/or ARB treatment passed adjudication. Almost all were of Swedish origin (93.1%), and there were more males than females (Table 2). One outlier among the cases was detected with principal component analysis, but not removed (Supplementary Fig. S4a, b). Most patients had a history of cardiovascular disease, including hypertension, angina pectoris, myocardial infarction or stroke, and approximately one in four suffered from diabetes. The angioedema event was in 144 patients associated with ACEi treatment, most frequently enalapril and ramipril. In 31 patients, the event was associated with an ARB, most frequently candesartan and losartan. Two patients developed angioedema during treatment with both an ACEi and an ARB (enalapril/candesartan and ramipril/losartan respectively). Frequent co-medications during the three months preceding the onset of angioedema were diuretics (47.4%), lipid-modifying agents (44.5%), beta-blockers (42.8%), antiplatelet drugs (42.2%), and selective calcium channel blockers (31.8%).

**Table 2:** Baseline characteristics of cases and treated controls in the discovery cohort.

	Cases (*N* = 173)	Controls (*N* = 1345)
Age at onset, mean (range)	65.6 [31–91]	68.9 [49–96]
Sex, *N* (%)		
Female	72 (41.6)	481 (36.0)
Male	101 (58.4)	864 (64.0)
Indications for treatment^a^, *N* (%)		
Hypertension	165 (95.4)	668 (69.9)
Diabetes	40 (23.1)	230 (24.1)
Heart failure	6 (3.4)	162 (16.9)
Type of ACEi^b^, *N* (%)		
C09AA02 Enalapril	116 (80.6)	1055 (78.4)
C09AA05 Ramipril	24 (16.7)	239 (17.8)
C09AA03 Lisinopril	3 (2.1)	27 (2.0)
C09AA01 Captopril	0 (0)	13 (1.0)
C09AA08 Cilazapril	0 (0)	8 (0.6)
C09AA06 Quinapril	1 (0.7)	1 (0.1)
C09AA09 Fosinopril	0 (0)	1 (0.1)
C09AA10 Trandolapril	0 (0)	1 (0.1)
Type of ARB^c^, *N* (%)		
C09CA06 Candesartan	12 (41.4)	N/A
C09CA01 Losartan	11 (37.9)	N/A
C09CA04 Irbesartan	4 (13.8)	N/A
C09CA07 Telmisartan	1 (3.4)	N/A
C09CA03 Valsartan	1 (3.4)	N/A

Drugs were categorised using the World Health Organization (WHO) Collaborating Centre for Drug Statistics Methodology International Anatomical Therapeutic Chemical (ATC) classification.

*ACEi* angiotensin-converting enzyme inhibitor, *ARB* angiotensin receptor blocker, *N* number of subjects, *N/A* not available.

^a^Data available for all 173 cases and for 956 of 1345 treated controls. Percentages are calculated using only individuals with data.

^b^Data available for all 144 ACEi-treated cases and for all 1345 of ACEi-treated controls. Two cases were treated with both an ACEi and an ARB.

^c^Data available for none of the controls and for all 29 ARB-treated cases.

Data from 4890 unrelated population controls were obtained from the Swedish Twin Registry [33]. The subgroup of treated controls (*n* = 1345) had collected at least two prescriptions of an ACEi at a Swedish Pharmacy without being diagnosed with angioedema or larynx-oedema (Table 2). Sex distribution among treated controls was similar to that of the cases with more males than females. Hypertension, diabetes and heart failure were common among the treated controls that had data on diagnoses (*n* = 956). The most frequently prescribed ACEis were enalapril and ramipril.

### Discovery cohort genome-wide analysis data analysis

**Fig. 1:**
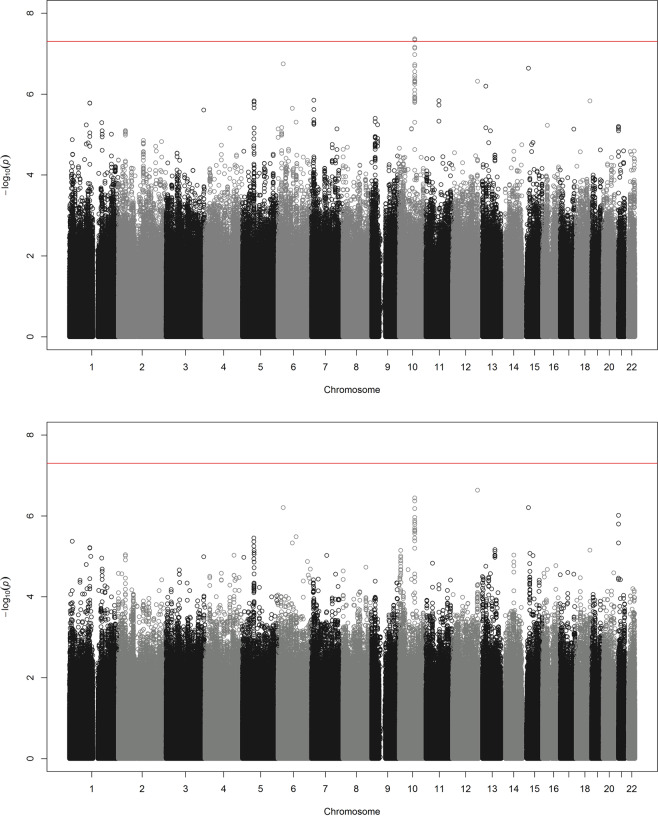
Manhattan plot of genome-wide association analysis. Analysis of 7585599 genome-wide single nucleotide polymorphisms in 173 cases of angioedema associated with angiotensin-converting enzyme inhibitors and/or angiotensin receptor blockers adjusted by sex and genetic principal components 1–4. The threshold for genome-wide significance (*p* < 5 × 10^−8^) is indicated by the red line on the *x*-axis where the scale is –log_10_(p). **a** Intronic SNPs in the *Calcium-activated potassium channel subunit alpha-1 (KCNMA1)* gene on chromosome 10 were significantly associated on a genome-wide level when cases were compared with all 4890 controls. **b** Associations were similar, but did not reach genome-wide significance, when cases were compared with 1345 controls matched for treatment.

**Fig. 2:**
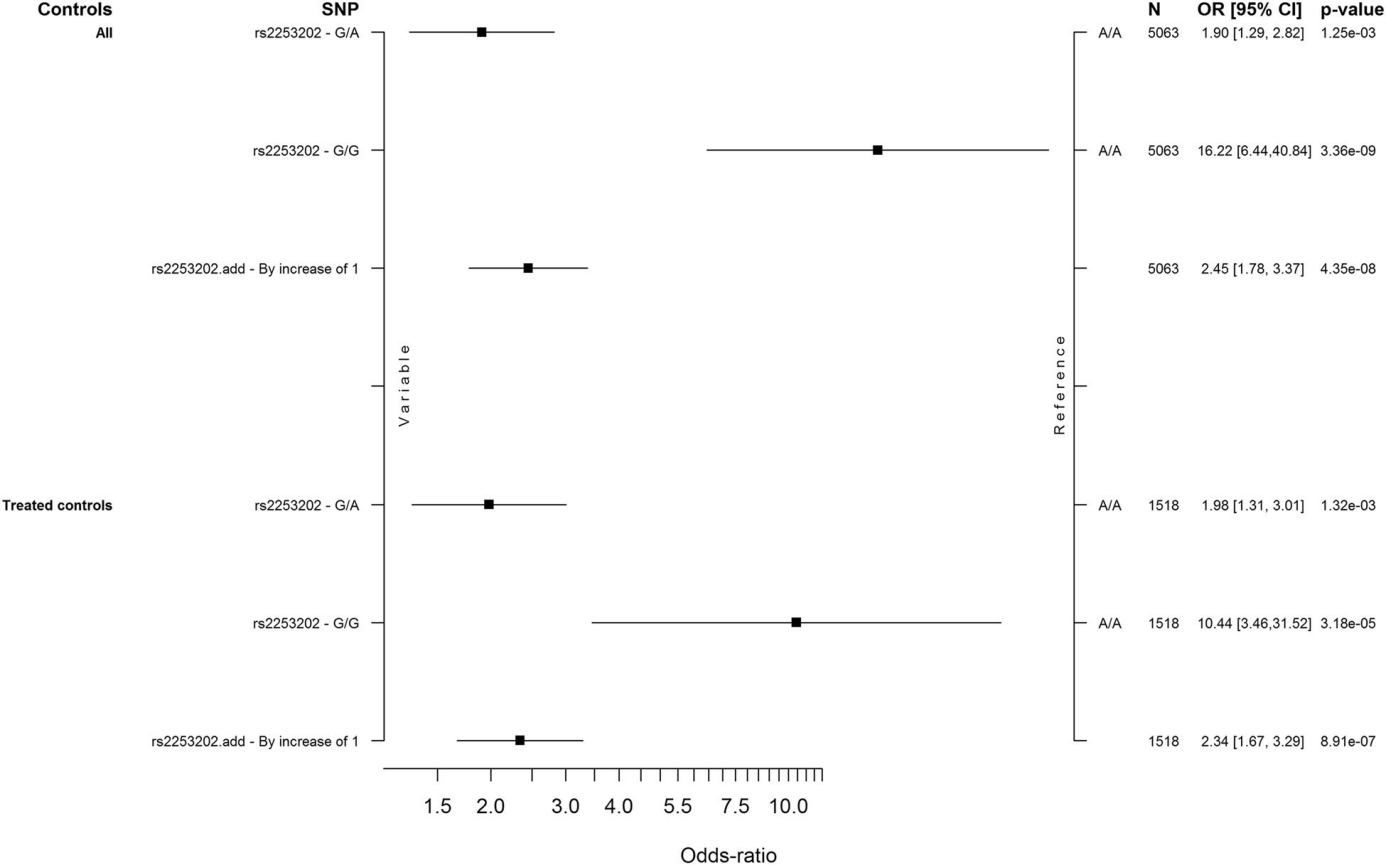
Estimated odds ratios for associations with rs2253202 in *KCNMA1*. A forest plot of the association with rs2253202 in the 173 discovery cases compared with all 4890 controls and with 1345 treated controls. Estimated odds ratios (OR) with 95% confidence intervals (CI), *p* value and numbers (N) included are shown. The effect is adjusted for sex and genetic principal components 1–4.

The calcium-activated potassium channel subunit alpha-1 (*KCNMA1*) gene on chromosome 10 was significantly associated with angioedema on a genome-wide level. The top hit in intron 1 of *KCNMA1* was rs2253201 (OR = 2.47, 95% CI = 1.79–3.42, *p* = 4.17 × 10^−8^), and several nearby SNPs had *p* values around 10^−7^ (Table 3a, Supplementary Table S1). After imputation, five additional SNPs in intron 1 of *KCNMA1* were associated on a genome-wide level: rs2253202 (OR = 2.47, 95% CI = 1.79–3.42, *p* = 4.31 × 10^−8^), rs2673471 (OR = 2.47, 95% CI = 1.79–3.41, *p* = 4.35 × 10^−8^) and rs2619635, rs2670121 and rs2673455 (all OR = 2.47, 95% CI = 1.79–3.41, *p* = 4.59 × 10^−8^) (Fig. 1a, Table 3b, Supplementary Table S2). Comparisons with the 1345 treated controls supported that the association with *KCNMA1* was not due to confounding by indication (OR = 2.34, 95% CI = 1.67–3.29, *p* = 8.91 × 10^−7^) (Figs. 1b and 2, Table 3c).

**Table 3:** Top ten genome-wide results in (a) genotyped single nucleotide polymorphisms (SNPs) using unmatched controls, (b) genotyped and imputed SNPs using unmatched controls, and (c) genotyped and imputed SNPs using matched controls.

CHR	SNP	BP	*N*	OR	L95	U95	*P*	GTPS	MAF case	MAF control	Gene
(a)											
10	rs2253201	79356397	5062	2.474	1.79	3.419	4.174E−08	G/A	0.136	0.060	*KCNMA1*
10	rs1949352	79249522	5062	2.399	1.744	3.299	7.372E−08	C/T	0.142	0.065	*KCNMA1*
10	rs2253202	79356393	5061	2.433	1.756	3.37	9.077E−08	G/A	0.134	0.060	*KCNMA1*
6	rs6913724	27254843	5062	1.804	1.446	2.252	1.788E−07	A/T	0.572	0.428	*POM121L2*
10	rs2255649	79343812	5063	2.387	1.721	3.312	1.865E−07	C/T	0.133	0.061	*KCNMA1*
10	rs1464111	79342926	5063	2.332	1.678	3.243	4.732E−07	T/C	0.130	0.061	*KCNMA1*
10	rs2670164	79327231	5057	2.342	1.679	3.266	5.392E−07	A/G	0.129	0.060	*KCNMA1*
10	rs816829	79263445	5062	2.269	1.628	3.162	1.306E−06	T/G	0128	0061	*KCNMA1*
10	rs816838	79274098	5058	2.258	1.621	3.146	1.488E−06	C/T	0127	0061	*KCNMA1*
10	rs1268510	79246052	5060	2.251	1.615	3.136	1.642E−06	A/G	0127	0061	*KCNMA1*
(b)											
10	rs2253201	79356397	5063	2.471	1.788	3.416	4.314E−08	G/A	0.136	0.060	*KCNMA1*
10	rs2253202	79356393	5063	2.471	1.788	3.416	4.314E−08	G/A	0.136	0.060	*KCNMA1*
10	rs2673471	79357323	5062	2.471	1.787	3.415	4.350E−08	A/G	0.136	0.060	*KCNMA1*
10	rs2619635	79358602	5063	2.467	1.785	3.41	4.592E−08	G/A	0.136	0.060	*KCNMA1*
10	rs2670121	79358889	5063	2.467	1.785	3.41	4.592E−08	A/G	0.136	0.060	*KCNMA1*
10	rs2673455	79359111	5063	2.467	1.785	3.41	4.592E−08	C/G	0.136	0.060	*KCNMA1*
10	rs865293	79258692	5062	2.402	1.747	3.304	6.979E−08	G/A	0.142	0.065	*KCNMA1*
10	rs1949352	79249522	5063	2.399	1.744	3.299	7.331E−08	C/T	0.142	0.065	*KCNMA1*
10	rs816847	79250577	5063	2.399	1.744	3.299	7.331E−08	G/C	0.142	0.065	*KCNMA1*
10	rs866539	79249804	5063	2.399	1.744	3.299	7.331E−08	C/T	0.142	0.065	*KCNMA1*
(c)											
12	rs77440358	128558842	1487	2.546	1.787	3.629	2.33E−07	T/C	0.144	0.065	Intergenic
10	rs865293	79258692	1517	2.405	1.715	3.373	3.61E−07	G/A	0.142	0.063	*KCNMA1*
10	rs1949352	79249522	1518	2.391	1.705	3.352	4.29E−07	C/T	0.142	0.064	*KCNMA1*
10	rs816847 G	79250577	1518	2.391	1.705	3.352	4.29E−07	G/C	0.142	0.064	*KCNMA1*
10	rs866539C	79249804	1518	2.391	1.705	3.352	4.29E−07	C/T	0.142	0.064	*KCNMA1*
15	rs11070816	31406335	1518	1.791	1.424	2.253	6.27E−07	T/A	0.569	0.414	*TRPM1*
6	rs6913724	27254843	1518	1.794	1.426	2.258	6.28E−07	A/T	0.572	0.424	*POM121L2*
10	rs2253201	79356397	1518	2.401	1.7	3.392	6.58E−07	G/A	0.136	0.06	*KCNMA1*
10	rs2253202	79356393	1518	2.401	1.7	3.392	6.58E−07	G/A	0.136	0.06	*KCNMA1*
10	rs2619635	79358602	1518	2.401	1.7	3.392	6.58E−07	G/A	0.136	0.06	*KCNMA1*

*CHR* chromosome, *BP* base pair (chromosome position), *N* number of study participants, *OR* odds ratio, *L95 and U95* lower and upper confidence interval, *P* *p* value, *MAF* minor allele frequency, *GTPS* genotypes minor/major allele, *E* *exponent* of 10.

### Discovery cohort candidate gene analysis

**Fig. 3:**
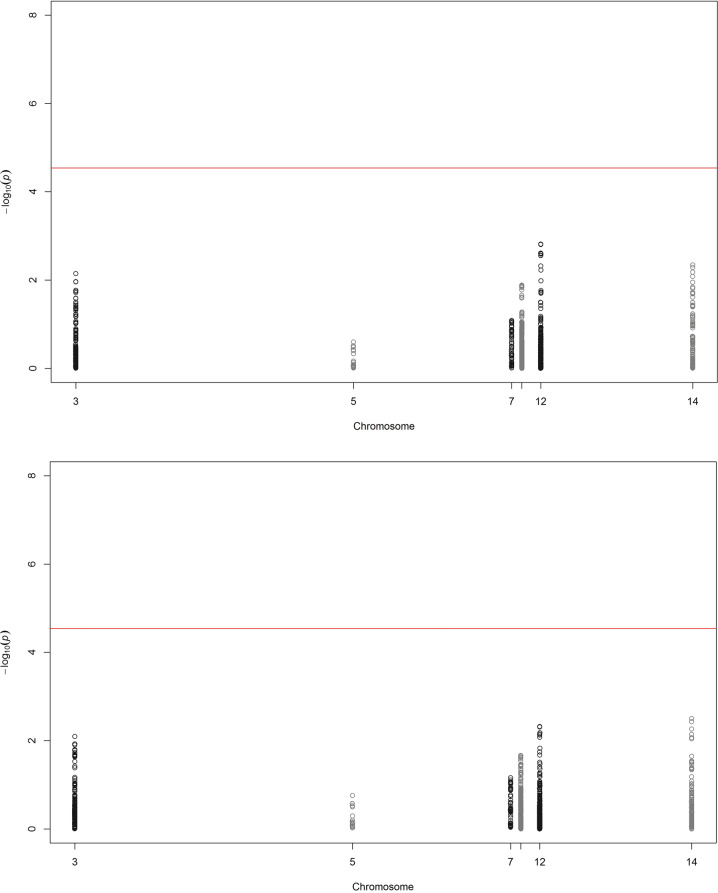
Manhattan plot of candidate gene association analysis. Analysis of 1730 candidate gene single nucleotide polymorphisms in 173 cases of angioedema associated with angiotensin-converting enzyme inhibitors and/or angiotensin receptor blockers adjusted by sex and genetic principal components 1–4. The threshold for candidate gene-wide significance (*p* < 2.89 × 10^−5^) is indicated by the red line on the *x*-axis where the scale is –log_10_(p). **a** None of the candidate genes *BDKRB2, ETV6, F12, MME, PRKCQ* or *SERPINE1* was significantly associated when cases were compared with all 4890 controls. **b** Results were consistent when cases were compared with 1345 controls matched for treatment.

*ETV6, BDKRB2, MME*, and *PRKCQ* were nominally associated with angioedema (*p* < 0.05), but none of the candidate genes was significantly associated after correction with multiple testing (*p* < 2.89 × 10^−5^) (Fig. 3a, Table 4a, Supplementary Table S3). Results were consistent when cases were compared with matched controls (Fig. 3b, Table 4b). The only published candidate SNPs present in our data were the presumed protective *PRKCQ* rs500766 and deleterious *MME* rs989692 [30]. Odds ratios for these SNPs were close to 1 in our study (Table 5).

**Table 4:** Top hits in imputed results for each of the candidate genes *ETV6, BDKRB2, MME, PRKCQ, SERPINE1*, and *F12* including 1000 bases upstream and downstream (a) when compared with unmatched controls, and (b) when compared with matched controls.

CHR	SNP	BP	*N*	OR	L95	U95	*P*	GTPS	MAF case	MAF control	Location	Gene
(a)												
12	rs113768037	12006062	5055	1.793	1.25	2.572	0.001525	C/A	0.108	0.065	Intron	*ETV6*
14	rs4905462	96680574	5046	1.455	1.123	1.885	0.00452	A/G	0.222	0.165	Intron	*BDKRB2*
3	rs3773896	154824685	5047	1.432	1.102	1.859	0.007161	C/A	0.222	0.168	Intron	*MME*
10	rs12784332	6471961	5063	0.7548	0.6046	0.9425	0.01302	T/C	0.387	0.452	Intron	*PRKCQ*
7	rs2227668	100774855	4991	0.7516	0.5446	1.037	0.0824	A/G	0.131	0.166	Intron	*SERPINE1*
5	rs4314418	176836426	4997	0.4384	0.1073	1.79	0.2506	A/C	0.006	0.013	Intron	*F12*
(b)												
14	rs4900315	96680903	1513	1.444	1.131	1.843	0.003166	C/T	0.341	0.268	Intron	*BDKRB2*
12	rs113768037	12006062	1514	1.747	1.186	2.573	0.004774	C/A	0.108	0.065	Intron	*ETV6*
3	rs3773896	154824685	1513	1.462	1.104	1.937	0.008019	C/A	0.222	0.165	Intron	*MME*
10	rs10906559	6475947	1507	0.764	0.6073	0.9612	0.02157	T/C	0.367	0.426	Intron	*PRKCQ*
7	rs7459187	100785438	1497	0.7151	0.4979	1.027	0.06941	T/C	0.109	0.145	Downstream	*SERPINE1*
5	rs4314418	176836426	1501	0.3722	0.08915	1.554	0.1754	A/C	0.006	0.016	Intron	*F12*

*CHR* chromosome, *BP* base pair (chromosome position), *N* number of study participants, *OR* odds ratio, *L95 and U95* lower and upper confidence interval, *P* p value, *MAF* minor allele frequency, *GTPS* genotypes minor/major allele, *E* *exponent* of 10.

**Table 5:** Results for candidate SNPs that previously have been associated with angiotensin-converting enzyme inhibitor-induced angioedema [30]. All cases are compared with unmatched controls.

CHR	SNP	BP	*N*	OR	L95	U95	*P*	GTPS	MAF case	MAF control	Gene
10	rs500766	6550590	5063	1.146	0.9074	1.446	0.2531	T/C	0.315	0.280	*PRKCQ*
3	rs989692	154801365	5063	0.975	0.7836	1.213	0.8199	T/C	0.468	0.484	*MME*

*CHR* chromosome, *BP* base pair (chromosome position), *N* number of study participants, *OR* odds ratio, *L95 and U95* lower and upper confidence interval, *P* *p* value, *MAF* minor allele frequency, *GTPS* genotypes minor/major allele, *E* *exponent* of 10.

### Replication cohort characteristics

The American cohort included 175 angioedema cases and 489 ACEi-exposed controls [30]. Sixty-six of the cases were of African, and 109 of European descent. Ethnic origin of the controls was 157 African, 330 European, and two unknown. The Northern European cohort included 238 angioedema cases and 110 treated controls. Specifically, there were 96 cases and 38 controls from Sweden, 60 cases and 20 controls from Denmark, 64 cases and 52 controls from the Netherlands, and 18 cases from the UK. All cases had been treated with an ACEi, and one also with an ARB. All controls were treated with an ACEi, and eight also with an ARB.

### Replication and meta-analysis

**Fig. 4:**
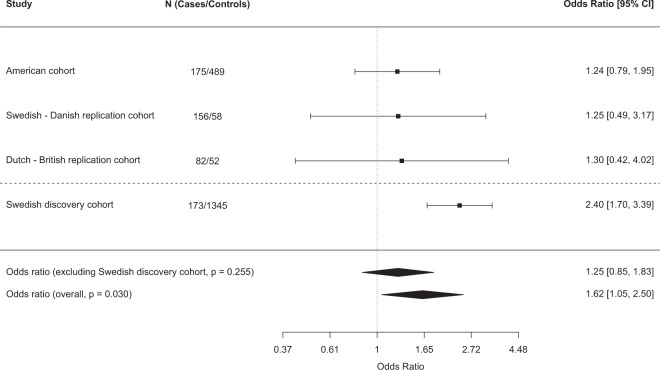
Meta-analysis. Meta-analysis of the top single nucleotide polymorphism rs2253202 in the replication cohorts from the US [30] and Northern Europe. Meta-analysis was performed with and without the Swedish discovery cohort using a fixed-effect model in the metafor R-package. Logistic regression was used to evaluate the association between the SNP and angioedema. SNP effects were modelled as additive. Estimated odds ratios with 95% confidence intervals (CI), *p* value and numbers (N) included are shown. The cut-off for a statistically significant association was set to *p* < 0.05.

We sought replication for the top hit rs2253202 in the American cohort [30] and the Northern European cohort divided into Swedish-Danish and Dutch-British (Fig. 3). All individual cohorts had odds ratios above 1, but the results did not reach significance. Meta-analysis of the 413 cases and 599 ACEi-exposed controls included in the replication likewise gave non-significant results (OR = 1.25, 95% CI = 0.85−1.83, *p* = 0.255) (Fig. 3). The discovery cohort was then added to the meta-analysis, making a grand total of 586 cases and 1944 ACEi-exposed controls, which rendered a statistically significant association between rs2253202 and angioedema (OR = 1.62, 95% CI = 1.05–2.50, *p* = 0.030) (Fig. 4). This means that the odds of experiencing angioedema increased 1.62 times per minor allele, i.e. that the risk would be (1.62)^2^ = 2.62 times increased in homozygous carriers of rs2253202.

### Functional analysis

The six significantly associated SNPs in the first intron of the *KCNMA1* gene are in a haplotype block spanning ∼0.1 Mb, and lack evidence of functional activity. They are in high LD (*r*^2^ > 0.8) with 15 non-coding SNPs located in enhancer elements defined in several angioedema relevant tissues such as skin, mucosa, oesophagus, aorta, visceral smooth muscle and endothelial cells (Supplementary Tables S4a, b, and Supplementary Fig. S5). According to motif analyses, eight of the SNPs alter binding sites for transcription factors enriched in skin, mucosa or smooth muscle (Supplementary Table S3b) [47]. Previous results from chromatin immunoprecipitation with massively parallel DNA sequencing (ChIP-seq) point to the SNP rs816827 G > A that overlaps binding sites of 22 transcription factors in different cell lines (Supplementary Fig. S6) [46, 47, 49]. Tissue-specific analyses show that rs816827 alters binding motifs for transcription factors enriched in enhancer elements in skin (orange), mucosa (pink) and smooth muscle (brown) (Supplementary Table S4b). The TRAP tool [49] indicates that the transcription factor MAF, which is expressed in for example skin, oral mucosa, oesophagus, aorta and smooth muscle, would bind significantly stronger to the minor than the major allele (difference log(p) for two alleles = −0.812 or A/*p* value = 0.0253 vs G/*p* value = 0.164).

## Discussion

This study aimed to identify common genetic risk factors for angioedema induced by ACEi or ARB treatment. Genetic variants in the calcium-activated potassium channel subunit alpha-1 (*KCNMA1*) gene were significantly associated with angioedema on a genome-wide level (*p* < 5 × 10^−8^). Calcium-activated potassium channel genes are expressed in vascular smooth muscle and endothelium, and encode effector proteins crucial for control of vascular tone and blood pressure [50]. We ascertained that our finding was not due to confounding by indication for treatment by redoing the analysis using only ACEi-treated controls. Furthermore, meta-analysis of four cohorts of cases and treated controls supported the association between *KCNMA1* and ACEi- or ARB-induced angioedema (Fig. 3).

The *KCNMA1* intron 1 variants significantly associated with angioedema in our study lack evidence of functional activity, but are in linkage disequilibrium with several genetic variants located in regulatory elements. Motif analyses indicate that one variant in particular alters binding sites for tissue-specific transcription factors, which could lead to deregulation of a target gene. The target gene could be distant, but it is more plausible that it is *KCNMA1* itself. *KCNMA1* encodes the pore-forming alpha subunit of the large-conductance calcium-dependent potassium big K (BK) channel, which is important for the repolarization of the cell membrane [50]. A study using an alpha subunit BK channel knockout mouse model suggested that this gene modulates the renin-angiotensin-aldosterone system [51]. *KCNMA1* is one among several genes considered to be involved in control of vascular tone and hypertension [50, 52]. A previous GWAS detected an association between a *KCNMA1* intron 1 SNP (rs603788, GRCh37 chr10: 79211262) and diastolic blood pressure in a subgroup of 3014 elderly patients (*p* = 9.37 × 10^−7^) [53]. In a candidate gene study, a *KCNMA1* intron 17 SNP (rs16934182, GRCh37 chr10: 78778734) was nominally associated with severe systolic hypertension (*p* = 0.03), and severe general hypertension (*p* = 0.04) [54]. These two intronic SNPs are not in LD with the six intron 1 variants significantly associated in our study (GRCh37 chr10:79356393-79359111) [55].

Genetic variants of calcium/potassium channel genes have previously been implicated in studies on ACEi-induced ADRs. A GWAS on ACEi-induced cough detected an association with the calcium-activated potassium channel gene *KCNMB2* [56], while another GWAS found an association with the calcium-binding potassium channel-interacting protein gene *KCNIP4* [57]. A clinical study showed that patients who reacted with angioedema to an ACEi were more often treated with a calcium channel blocker than those who reacted with cough [58]. Furthermore, angioedema is in rare cases triggered by a calcium channel blocker (e.g. amlodipine) or potassium channel blocker (e.g. amiodarone) alone [59, 60]. It is thus reasonable to believe that deficient transport of calcium and/or potassium across the cell membrane plays a role in the development of angioedema.

In addition, we analysed data for the candidate genes *BDKRB2, ETV6, F12, MME, PRKCQ*, and *SERPINE1*. There was no significant association with single nucleotide polymorphisms in the candidate genes after correcting for multiple testing. *ETV6* showed promising results with relatively high odds ratios and *p* values around 0.001. A larger sample size would be needed to determine whether any candidate gene is truly associated with angioedema induced by ACEi or ARB treatment.

A strength of this study is that the cases were well-characterised and the diagnostic accuracy was high. There are also limitations. First, matching of controls was done using ACEi exposure as a proxy for the indication for treatment. We were thus unable to identify whether treated controls were prescribed ACEis for hypertension, which was the most common diagnosis among cases. Secondly, although this is the largest GWAS on ACEi- or ARB-induced angioedema in people of European ancestry to date, the low number of cases does not provide enough power to detect association with rare variants. Nor could we show whether the trait in question is affected by multiple weakly associated variants that interact to increase the risk.

Our GWAS suggests that common genetic variants of *KCNMA1* are associated with risk of ACEi- or ARB-induced angioedema. Comparisons between cases and treated controls confirm that the finding is not due to confounding by indication. Meta-analysis of replication cohorts from the US [30] and Northern Europe together with the discovery cohort further supports the association. This is a step forward in the understanding of mechanisms behind ACEi- or ABR-induced angioedema. It is, however, reasonable to believe that intron 1 of *KCNMA1* is only one of several susceptibility loci. Future studies based on whole exome or genome sequencing will show whether rare coding variants contribute to the risk of ACEi- or ABR-induced angioedema to a clinically important degree. When the complete picture is known, and patients have DNA sequence data available in the medical record before treatment, personalised medical decisions can be used to prevent this potentially fatal ADR.

## Supplementary information

Supplemental material
