# Supplementary material for: Genome-wide association study of angioedema induced by angiotensin-converting enzyme inhibitor and angiotensin receptor blocker treatment
Source: Pharmacogenomics J. 2020 Feb 21;20(6):770–83. doi: 10.1038/s41397-020-0165-2 (PMC7674154; doi:10.1038/s41397-020-0165-2)
Supplement: Supplementary file 1 — Supplemental material [file 41397_2020_165_MOESM1_ESM.docx]

**Supplement to Genome-wide association study of angioedema induced by angiotensin-converting enzyme inhibitor and angiotensin receptor blocker treatment**

Subjects and Methods describes the ethics approvals and cohorts in detail. Tables are the 100 most associated genotyped and imputed variants, and chromatin states and functional annotations. Figures are of power calculation, Q-Q plot, principal component analyses, and linkage disequilibrium and genomic and transcription factor background of the *KCNMA1* variant.

**Subjects and Methods**

**Ethical statement**

Research was carried out in accordance with the latest update of the Declaration of Helsinki. Written informed consent was obtained from all participants. The study protocols were approved by Regional Ethics Committees in Uppsala (Dnr 2010/231) and Stockholm (Dnr 2007-644-31 and 2011/463-32) in Sweden; the Danish Data Protection Agency (Journal number 2008-58-0035) and Ethics Committee (ID S-20140165) in Denmark; the Central Medical Ethical Committees of the Medical Centre Vrije Universiteit (VU) Amsterdam and University Medical Center (UMC) Utrecht, and local Medical Ethical Committees of each participating hospital in the Netherlands; the East of Scotland Ethics Approval Board and National Health Service Tayside Caldicott, the Sefton Research Ethics Committee (IRAS ID: 7086), the Liverpool (Adult) Research Ethics Committee (IRAS ID: 31492), the North-West Research Ethics Committee - Liverpool Central, and local ethic committees at Royal Liverpool Hospital and Southampton General Hospital in the UK. The Institutional Review Boards of Vanderbilt and Marshfield had approved the American study that was used in the meta-analysis.^1^

**Discovery cohort description**

Angioedema cases were recruited through SWEDEGENE (www.swedegene.se). All cases associated with angiotensin-converting enzyme inhibitor (ACEi) or angiotensin receptor blocker (ARB) treatment reported to the Swedish national ADR registry at the Medical Products Agency January 1990 - March 2016 were retrieved. Patients at least 18 years of age and considered suitable by the reporting physician were contacted. A minority of patients were referred directly from collaborating clinicians. Inclusion and exclusion criteria were according to published phenotype standardisation criteria.^2^ In brief, the initial event should have occurred during treatment with an ACEi or ARB, and be documented in the medical record. Consenting patients were interviewed by telephone regarding the ADR, concomitant medications, disease history, patient demographics, life-style factors and relevant family history. Patient medical records were retrieved to complement the medical information. The suspected drug should have been discontinued, but it was acceptable if the angioedema recurred within one year of stopping the drug. Concomitant drugs were categorized using the World Health Organization (WHO) Collaborating Centre for Drug Statistics Methodology International Anatomical Therapeutic Chemical (ATC) classification.^3^ Exclusion criteria were concomitant urticaria, another suspected cause of angioedema or a history of hereditary angioedema or acquired complement C1-esterase inhibitor deficiency. In addition, cases with a cognitive impairment were excluded. All phenotype data were entered into a database, were reviewed and adjudicated by a clinical expert in allergology. Patients provided an EDTA blood sample that was stored at -70°C until DNA extraction according to standard procedures.

Unrelated population controls were obtained from the Swedish Twin registry. The subgroup of treated controls had collected at least two prescriptions of an ACEi at a Swedish pharmacy between 2005 and 2012 according to the Swedish Prescribed Drug Register.^4,5^ Indications for treatment was searched for in the Swedish National Patient Register using International Statistical Classification of Diseases and Related Health Problems 10 (ICD-10) codes for hypertension (I10-I15), heart failure (I50), and diabetes (E10-E14).^6^ Indications were only available for 71% of the controls, since the register has a low sensitivity for primary care diagnosis.^7^ Controls who had the diagnoses angioedema or larynx-oedema in the Swedish National Patient Register were excluded.^6^ The diagnoses angioedema and larynx-oedema were defined using ICD-9 codes 9951 and 4786, and ICD-10 codes T783 and J384.^8^

**Replication cohort description**

Data from the published ACEi-angiodema GWAS cohort from Vanderbilt and Marshfield, USA, was obtained through collaboration.^1^ Informed consent had been obtained from all participants. Cases were defined as patients who developed swelling of the lips, pharynx or face for the first time while taking an ACE inhibitor. All patients had been interviewed by a physician or research nurse, and their medical history was confirmed using a case report form. Controls had been treated with an ACEi for at least 6 months without developing angioedema.

In Sweden, patients for the replication were recruited April 2016 - March 2017 in the same way as the discovery cohort. In Denmark, patients were referred May 2015 - September 2016 by physicians at Roskilde Hospital, University Hospital of Copenhagen (Rigshospitalet), Gentofte Hospital, University Hospital of Zealand, Slagelse Hospital, Bispebjerg Hospital, University Hospital of Odense and by a collaborating general practitioner. In the Netherlands, patient recruitment took place December 2013 - February 2017 at the VU Amsterdam Medical Centre, Academic Medical Centre (AMC) Amsterdam, University Medical Centre (UMC) Groningen, Westfriesgasthuis in Hoorn, Noordwest Hospital Group, UMC Utrecht and UMC Maastricht. Patients were referred directly from physicians at the participating hospitals or identified by screening electronic medical records from all hospital admissions for angioedema during a 10-year period. In the UK, patients were identified December 2014 - October 2015 by direct referral from two consultant immunologists at Royal Liverpool Hospital, Southampton General Hospital and a consultant immunologist at Ninewells Hospital and Medical School in Dundee. Furthermore, patients were identified by review of archived clinical letters at Ninewells Hospital.

Patients were approached by telephone and/or received an invitation letter. Participants signed an informed consent form, and were provided with a patient information leaflet and a questionnaire surveying demographics, causative drug, symptoms of angioedema, other possible triggers, family history of angioedema, co-medications around the time of angioedema and a few items of medical history. When needed, medical records were used to complete the questionnaire. The expert who assessed the discovery cases also assessed the replication cases and used identical criteria.^2^

Treated controls from Sweden were recruited by collaborating clinicians. Danish treated controls were selected from existing databases.^9^ Dutch treated controls were participants of Utrecht Cardiovascular Pharmacogenomics (UCP) studies whose drug dispensing data and hospital discharge diagnoses were obtained through the Dutch population-based Pharmaco-Morbidity Record Linkage System (PHARMO) database.^10^ British treated controls were patients in the Genetics of Diabetes Audit and Research Tayside Study (GoDARTS) who had filled continuous ACEi prescriptions for at least one year without an ICD-9 or ICD-10 code for the diagnosis angioedema.^11^ All patients and controls provided a blood sample as above or saliva sample (2 ml Oragene® OG-500 collection kit, DNA Genotek, Canada). Blood samples were kept at -70°C and saliva samples at room temperature until DNA extraction. DNA was extracted according to standard procedures.

**Table S1.** The 100 genotyped variants with the highest association with angioedema induced by angiotensin-converting enzyme inhibitor (ACEi) or angiotensin receptor blocker (ARB) treatment when cases were compared with all controls.

| **CHR** | **SNP** | **BP** | **N** | **OR** | **L95** | **U95** | **P** | **MAF** | **GTPS** |
| --- | --- | --- | --- | --- | --- | --- | --- | --- | --- |
| **10** | rs2253201 | 79356397 | 5062 | 2.474 | 1.79 | 3.419 | 4.174e-08 | 0.063 | G/A |
| **10** | rs1949352 | 79249522 | 5062 | 2.399 | 1.744 | 3.299 | 7.372e-08 | 0.068 | C/T |
| **10** | rs2253202 | 79356393 | 5061 | 2.433 | 1.756 | 3.37 | 9.077e-08 | 0.063 | G/A |
| **6** | rs6913724 | 27254843 | 5062 | 1.804 | 1.446 | 2.252 | 1.788e-07 | 0.432 | A/T |
| **10** | rs2255649 | 79343812 | 5063 | 2.387 | 1.721 | 3.312 | 1.865e-07 | 0.064 | C/T |
| **10** | rs1464111 | 79342926 | 5063 | 2.332 | 1.678 | 3.243 | 4.732e-07 | 0.063 | T/C |
| **10** | rs2670164 | 79327231 | 5057 | 2.342 | 1.679 | 3.266 | 5.392e-07 | 0.063 | A/G |
| **10** | rs816829 | 79263445 | 5062 | 2.269 | 1.628 | 3.162 | 1.306e-06 | 0.063 | T/G |
| **10** | rs816838 | 79274098 | 5058 | 2.258 | 1.621 | 3.146 | 1.488e-06 | 0.063 | C/T |
| **10** | rs1268510 | 79246052 | 5060 | 2.251 | 1.615 | 3.136 | 1.642e-06 | 0.064 | A/G |
| **10** | rs2395479 | 79318095 | 5058 | 2.214 | 1.578 | 3.105 | 4.164e-06 | 0.062 | T/C |
| **2** | rs7569878 | 36559139 | 5063 | 3.234 | 1.93 | 5.419 | 8.387e-06 | 0.017 | T/C |
| **13** | rs1711774 | 60089243 | 5042 | 0.58 | 0.4563 | 0.7371 | 8.477e-06 | 0.403 | T/G |
| **1** | rs17344586 | 167244771 | 5063 | 2.066 | 1.498 | 2.849 | 9.814e-06 | 0.07 | A/G |
| **1** | rs2936014 | 214550568 | 5063 | 1.764 | 1.372 | 2.269 | 9.829e-06 | 0.172 | G/A |
| **9** | rs12554111 | 18424272 | 5054 | 1.688 | 1.332 | 2.14 | 1.501e-05 | 0.214 | G/T |
| **2** | rs12469732 | 127653376 | 5062 | 2.02 | 1.467 | 2.781 | 1.627e-05 | 0.076 | T/C |
| **5** | rs13182982 | 59038610 | 5052 | 2.121 | 1.505 | 2.99 | 1.756e-05 | 0.056 | G/A |
| **9** | rs947144 | 18653380 | 5062 | 1.688 | 1.329 | 2.145 | 1.775e-05 | 0.202 | T/C |
| **6** | rs1058768 | 54186147 | 5062 | 1.602 | 1.286 | 1.996 | 2.572e-05 | 0.337 | T/C |
| **2** | rs17743968 | 127659845 | 5060 | 2.019 | 1.452 | 2.808 | 2.986e-05 | 0.071 | T/C |
| **3** | rs6793348 | 9114759 | 5056 | 1.65 | 1.301 | 2.094 | 3.705e-05 | 0.211 | C/T |
| **17** | rs12452527 | 108420 | 5060 | 0.5983 | 0.4682 | 0.7646 | 4.032e-05 | 0.371 | A/G |
| **13** | rs7990587 | 83185044 | 5062 | 1.566 | 1.264 | 1.941 | 4.165e-05 | 0.367 | T/C |
| **22** | rs6009669 | 49807696 | 5063 | 0.5419 | 0.4037 | 0.7274 | 4.53e-05 | 0.255 | C/T |
| **6** | rs1508642 | 54185864 | 5059 | 1.576 | 1.266 | 1.963 | 4.802e-05 | 0.332 | G/A |
| **8** | rs4961172 | 87313475 | 5044 | 1.576 | 1.264 | 1.964 | 5.331e-05 | 0.446 | C/T |
| **2** | rs1438134 | 23512029 | 5041 | 1.595 | 1.271 | 2.002 | 5.513e-05 | 0.264 | G/A |
| **10** | rs1925546 | 20966815 | 5059 | 1.576 | 1.263 | 1.966 | 5.567e-05 | 0.489 | A/G |
| **20** | rs7263518 | 42829691 | 5062 | 1.735 | 1.327 | 2.268 | 5.713e-05 | 0.142 | T/C |
| **8** | rs13265072 | 14423424 | 5061 | 1.568 | 1.258 | 1.955 | 6.173e-05 | 0.335 | C/A |
| **2** | rs2577732 | 23507017 | 5062 | 1.589 | 1.267 | 1.994 | 6.226e-05 | 0.264 | T/C |
| **10** | rs4749197 | 7767196 | 5059 | 1.627 | 1.282 | 2.065 | 6.266e-05 | 0.204 | T/G |
| **8** | rs2100485 | 2695795 | 5060 | 1.832 | 1.361 | 2.467 | 6.636e-05 | 0.097 | G/T |
| **1** | rs2067795 | 234264472 | 5061 | 2.387 | 1.556 | 3.662 | 6.816e-05 | 0.033 | A/G |
| **6** | rs7740158 | 19229514 | 5061 | 3.215 | 1.81 | 5.713 | 6.821e-05 | 0.015 | C/T |
| **3** | rs7636574 | 56871432 | 5060 | 1.612 | 1.273 | 2.041 | 7.408e-05 | 0.227 | A/G |
| **10** | rs11592131 | 29216834 | 5055 | 2.042 | 1.433 | 2.91 | 7.725e-05 | 0.06 | C/T |
| **2** | rs7560096 | 31061536 | 5042 | 1.572 | 1.256 | 1.966 | 7.73e-05 | 0.258 | A/G |
| **7** | rs1991763 | 13469277 | 5062 | 1.581 | 1.26 | 1.984 | 7.766e-05 | 0.286 | G/A |
| **1** | rs3013446 | 214558228 | 5063 | 1.686 | 1.3 | 2.186 | 8.128e-05 | 0.164 | A/G |
| **6** | rs747199 | 44194345 | 5060 | 1.628 | 1.277 | 2.076 | 8.524e-05 | 0.185 | C/G |
| **1** | rs3912023 | 11630122 | 5052 | 1.799 | 1.342 | 2.411 | 8.635e-05 | 0.104 | T/C |
| **2** | rs4663057 | 127646013 | 5063 | 1.793 | 1.34 | 2.4 | 8.653e-05 | 0.11 | A/G |
| **2** | rs6714117 | 67114505 | 5063 | 1.886 | 1.374 | 2.59 | 8.754e-05 | 0.079 | C/T |
| **7** | rs4717428 | 67464503 | 5063 | 1.666 | 1.291 | 2.15 | 8.876e-05 | 0.163 | G/A |
| **10** | rs2026609 | 7751626 | 5062 | 1.61 | 1.269 | 2.042 | 8.935e-05 | 0.206 | G/A |
| **7** | rs17610533 | 138832936 | 5061 | 2.998 | 1.731 | 5.192 | 8.986e-05 | 0.016 | C/T |
| **2** | rs4665585 | 23516743 | 5062 | 1.572 | 1.253 | 1.972 | 9.157e-05 | 0.264 | G/A |
| **5** | rs13168097 | 59178177 | 5062 | 2.135 | 1.459 | 3.124 | 9.417e-05 | 0.045 | C/T |
| **8** | rs17761609 | 2701836 | 5061 | 1.787 | 1.335 | 2.391 | 9.472e-05 | 0.106 | G/A |
| **10** | rs7100211 | 3727947 | 5063 | 0.4658 | 0.3173 | 0.6837 | 9.56e-05 | 0.165 | C/T |
| **8** | rs16886089 | 115553747 | 5052 | 2.22 | 1.485 | 3.32 | 0.0001023 | 0.04 | A/C |
| **3** | rs13081907 | 94916211 | 5056 | 1.596 | 1.261 | 2.021 | 0.0001026 | 0.216 | G/A |
| **5** | rs6895849 | 156906819 | 5063 | 2.128 | 1.453 | 3.118 | 0.0001056 | 0.046 | T/C |
| **1** | rs10864520 | 11633982 | 5063 | 1.768 | 1.325 | 2.359 | 0.0001071 | 0.109 | G/A |
| **19** | rs12462536 | 45690775 | 5057 | 1.832 | 1.348 | 2.488 | 0.0001073 | 0.09 | G/A |
| **1** | rs4920653 | 17366871 | 5061 | 1.585 | 1.256 | 2.002 | 0.0001078 | 0.248 | C/T |
| **7** | rs7808481 | 10297082 | 5063 | 2.974 | 1.713 | 5.164 | 0.0001081 | 0.017 | A/G |
| **16** | rs4238574 | 20141365 | 5055 | 1.576 | 1.252 | 1.983 | 0.0001087 | 0.26 | C/T |
| **10** | rs816846 | 79279315 | 5062 | 1.669 | 1.288 | 2.164 | 0.0001094 | 0.162 | T/C |
| **7** | rs2908572 | 120724454 | 5054 | 1.71 | 1.302 | 2.246 | 0.0001136 | 0.133 | C/T |
| **21** | rs2826587 | 22255696 | 5062 | 1.623 | 1.269 | 2.077 | 0.0001149 | 0.19 | G/A |
| **6** | rs1546965 | 39999149 | 5063 | 0.5688 | 0.427 | 0.7578 | 0.0001159 | 0.264 | C/T |
| **6** | rs847744 | 40085245 | 5060 | 0.5705 | 0.4285 | 0.7596 | 0.0001217 | 0.263 | C/T |
| **9** | rs914708 | 132196531 | 5059 | 0.6471 | 0.5182 | 0.808 | 0.0001227 | 0.494 | G/T |
| **16** | rs2967392 | 82230815 | 5059 | 2.636 | 1.607 | 4.322 | 0.0001228 | 0.024 | C/T |
| **6** | rs3857470 | 94015959 | 5063 | 1.993 | 1.401 | 2.834 | 0.0001238 | 0.057 | T/C |
| **2** | rs2288353 | 145187783 | 5057 | 1.554 | 1.24 | 1.948 | 0.0001275 | 0.308 | C/T |
| **20** | rs8268 | 42825929 | 5062 | 1.712 | 1.3 | 2.254 | 0.0001281 | 0.136 | T/C |
| **9** | rs10987149 | 128857851 | 5062 | 0.5995 | 0.461 | 0.7796 | 0.0001343 | 0.314 | G/A |
| **16** | rs11074680 | 25245980 | 5063 | 1.664 | 1.281 | 2.162 | 0.0001368 | 0.154 | A/G |
| **20** | rs4364072 | 43013966 | 5061 | 1.542 | 1.234 | 1.926 | 0.0001368 | 0.3 | G/A |
| **1** | rs2746543 | 17386880 | 5057 | 1.557 | 1.24 | 1.955 | 0.0001369 | 0.284 | G/A |
| **11** | rs1493745 | 4724680 | 5063 | 3.205 | 1.761 | 5.835 | 0.000138 | 0.014 | A/G |
| **11** | rs16933260 | 4725667 | 5063 | 3.205 | 1.761 | 5.835 | 0.000138 | 0.014 | T/C |
| **11** | rs7478759 | 4724486 | 5063 | 3.205 | 1.761 | 5.835 | 0.000138 | 0.014 | T/C |
| **9** | rs752757 | 116122954 | 5063 | 1.629 | 1.268 | 2.095 | 0.0001387 | 0.169 | G/A |
| **19** | rs563320 | 8517386 | 5062 | 0.6101 | 0.4732 | 0.7867 | 0.0001389 | 0.335 | A/C |
| **1** | rs4655040 | 22638050 | 5063 | 1.53 | 1.229 | 1.904 | 0.0001393 | 0.336 | G/A |
| **7** | rs319870 | 78229176 | 5062 | 1.559 | 1.24 | 1.96 | 0.0001428 | 0.261 | G/A |
| **2** | rs17042334 | 668685 | 5061 | 1.63 | 1.267 | 2.098 | 0.000143 | 0.168 | T/C |
| **3** | rs2290055 | 108549720 | 5062 | 0.5426 | 0.3959 | 0.7437 | 0.0001438 | 0.221 | G/T |
| **6** | rs847769 | 40050171 | 5060 | 0.574 | 0.431 | 0.7643 | 0.0001453 | 0.263 | C/T |
| **6** | rs1240825 | 16862985 | 5061 | 2.023 | 1.405 | 2.913 | 0.0001509 | 0.056 | T/C |
| **8** | rs4738099 | 56282834 | 5063 | 1.531 | 1.228 | 1.908 | 0.0001514 | 0.481 | T/G |
| **16** | rs12920152 | 55278424 | 5058 | 1.724 | 1.3 | 2.286 | 0.0001537 | 0.128 | A/G |
| **18** | rs7237386 | 51180715 | 5061 | 0.592 | 0.4512 | 0.7767 | 0.0001542 | 0.297 | C/T |
| **10** | rs4748699 | 20984907 | 5063 | 1.674 | 1.282 | 2.187 | 0.0001545 | 0.136 | A/G |
| **20** | rs17753384 | 22371671 | 5061 | 2.173 | 1.453 | 3.248 | 0.000155 | 0.043 | G/A |
| **2** | rs4854351 | 661645 | 5038 | 1.621 | 1.262 | 2.084 | 0.0001594 | 0.168 | A/G |
| **3** | rs9968060 | 62471282 | 5055 | 1.523 | 1.224 | 1.895 | 0.0001599 | 0.35 | C/T |
| **10** | rs10828105 | 20970307 | 5060 | 1.676 | 1.282 | 2.192 | 0.0001605 | 0.139 | A/G |
| **15** | rs7177932 | 71720944 | 5062 | 0.5659 | 0.4208 | 0.761 | 0.0001651 | 0.246 | A/G |
| **2** | rs10497741 | 193920436 | 5063 | 1.976 | 1.386 | 2.817 | 0.0001671 | 0.056 | C/A |
| **2** | rs6737070 | 127665641 | 5059 | 1.75 | 1.307 | 2.342 | 0.0001688 | 0.113 | A/G |
| **2** | rs772781 | 235242143 | 5061 | 1.52 | 1.222 | 1.891 | 0.0001693 | 0.361 | C/T |
| **10** | rs10828101 | 20965972 | 5055 | 1.673 | 1.279 | 2.187 | 0.0001711 | 0.14 | A/G |
| **18** | rs8095679 | 58105704 | 5063 | 1.847 | 1.341 | 2.544 | 0.000173 | 0.083 | A/C |
| **3** | rs7636889 | 56874033 | 5062 | 1.571 | 1.241 | 1.989 | 0.0001743 | 0.23 | G/A |

CHR = chromosome, BP = base pair (chromosome position), N = number of study participants, OR = odds ratio, L95 and U95 = lower and upper confidence interval, P = p-value, MAF = Minor allele frequency, GTPS = Genotypes minor/major allele, E = exponent of 10.

**Table S2.** The 100 imputed variants with the highest association with angioedema induced by angiotensin-converting enzyme inhibitor (ACEi) or angiotensin receptor blocker (ARB) treatment when cases were compared with all controls.

| **CHR** | **SNP** | **BP** | **N** | **OR** | **L95** | **U95** | **P** | **MAF** | **GTPS** |
| --- | --- | --- | --- | --- | --- | --- | --- | --- | --- |
| **10** | rs2253201 | 79356397 | 5063 | 2.471 | 1.788 | 3.416 | 4.314e-08 | 0.063 | G/A |
| **10** | rs2253202 | 79356393 | 5063 | 2.471 | 1.788 | 3.416 | 4.314e-08 | 0.063 | G/A |
| **10** | rs2673471 | 79357323 | 5062 | 2.471 | 1.787 | 3.415 | 4.35e-08 | 0.063 | A/G |
| **10** | rs2619635 | 79358602 | 5063 | 2.467 | 1.785 | 3.41 | 4.592e-08 | 0.063 | G/A |
| **10** | rs2670121 | 79358889 | 5063 | 2.467 | 1.785 | 3.41 | 4.592e-08 | 0.063 | A/G |
| **10** | rs2673455 | 79359111 | 5063 | 2.467 | 1.785 | 3.41 | 4.592e-08 | 0.063 | C/G |
| **10** | rs865293 | 79258692 | 5062 | 2.402 | 1.747 | 3.304 | 6.979e-08 | 0.068 | G/A |
| **10** | rs1949352 | 79249522 | 5063 | 2.399 | 1.744 | 3.299 | 7.331e-08 | 0.068 | C/T |
| **10** | rs816847 | 79250577 | 5063 | 2.399 | 1.744 | 3.299 | 7.331e-08 | 0.068 | G/C |
| **10** | rs866539 | 79249804 | 5063 | 2.399 | 1.744 | 3.299 | 7.331e-08 | 0.068 | C/T |
| **10** | rs2673402 | 79357707 | 5061 | 2.421 | 1.748 | 3.354 | 1.054e-07 | 0.062 | T/A |
| **6** | rs6913724 | 27254843 | 5063 | 1.804 | 1.446 | 2.252 | 1.787e-07 | 0.432 | A/T |
| **10** | rs2255649 | 79343812 | 5063 | 2.387 | 1.721 | 3.312 | 1.865e-07 | 0.064 | C/T |
| **10** | rs2255656 | 79343778 | 5063 | 2.387 | 1.721 | 3.312 | 1.865e-07 | 0.064 | C/A |
| **10** | rs2619609 | 79336867 | 5063 | 2.381 | 1.716 | 3.303 | 2.041e-07 | 0.064 | C/T |
| **15** | rs11070816 | 31406335 | 5063 | 1.778 | 1.43 | 2.212 | 2.289e-07 | 0.425 | T/A |
| **10** | rs2253530 | 79353106 | 5029 | 2.393 | 1.716 | 3.339 | 2.775e-07 | 0.059 | T/G |
| **10** | rs2253545 | 79352739 | 5029 | 2.393 | 1.716 | 3.339 | 2.775e-07 | 0.059 | T/C |
| **10** | rs2619615 | 79352202 | 5029 | 2.393 | 1.716 | 3.339 | 2.775e-07 | 0.059 | T/A |
| **10** | rs2670146 | 79331412 | 5054 | 2.358 | 1.695 | 3.279 | 3.474e-07 | 0.063 | A/G |
| **10** | rs2719980 | 79330796 | 5054 | 2.358 | 1.695 | 3.279 | 3.474e-07 | 0.063 | G/A |
| **10** | rs2719981 | 79331919 | 5054 | 2.358 | 1.695 | 3.279 | 3.474e-07 | 0.063 | G/A |
| **10** | rs2619638 | 79319609 | 5063 | 2.346 | 1.687 | 3.263 | 3.971e-07 | 0.063 | A/G |
| **10** | rs2670108 | 79318855 | 5063 | 2.346 | 1.687 | 3.263 | 3.971e-07 | 0.063 | T/C |
| **10** | rs56254932 | 79316672 | 5063 | 2.346 | 1.687 | 3.263 | 3.971e-07 | 0.063 | T/TCCTAGGACCCCTGC... |
| **10** | rs1248571 | 79316781 | 5054 | 2.335 | 1.68 | 3.248 | 4.6e-07 | 0.063 | G/A |
| **10** | rs1464111 | 79342926 | 5063 | 2.332 | 1.678 | 3.243 | 4.732e-07 | 0.063 | T/C |
| **10** | rs2243681 | 79343161 | 5063 | 2.332 | 1.678 | 3.243 | 4.732e-07 | 0.063 | C/T |
| **12** | rs77440358 | 128558842 | 4974 | 2.267 | 1.648 | 3.118 | 4.813e-07 | 0.073 | T/C |
| **10** | rs2619613 | 79339707 | 5063 | 2.329 | 1.675 | 3.238 | 4.945e-07 | 0.063 | G/A |
| **10** | rs2244338 | 79337328 | 5062 | 2.329 | 1.675 | 3.238 | 4.971e-07 | 0.063 | C/T |
| **10** | rs2619610 | 79337132 | 5062 | 2.329 | 1.675 | 3.238 | 4.971e-07 | 0.063 | G/A |
| **10** | rs2673416 | 79336594 | 5063 | 2.326 | 1.673 | 3.234 | 5.162e-07 | 0.063 | T/C |
| **10** | rs2673457 | 79334952 | 5063 | 2.326 | 1.673 | 3.234 | 5.162e-07 | 0.063 | G/A |
| **10** | rs2670117 | 79354471 | 5051 | 2.33 | 1.673 | 3.247 | 5.747e-07 | 0.062 | A/G |
| **10** | rs2673429 | 79350798 | 5051 | 2.33 | 1.673 | 3.247 | 5.747e-07 | 0.062 | A/G |
| **10** | rs2928137 | 79352552 | 5051 | 2.33 | 1.673 | 3.247 | 5.747e-07 | 0.062 | A/C |
| **13** | rs113325073 | 36517430 | 4968 | 3.969 | 2.307 | 6.828 | 6.405e-07 | 0.013 | A/C |
| **10** | rs2619639 | 79319678 | 5061 | 2.315 | 1.659 | 3.231 | 7.939e-07 | 0.062 | A/G |
| **10** | rs111952376 | 79314815 | 5063 | 2.291 | 1.644 | 3.194 | 9.908e-07 | 0.063 | TACACACACACACACACACAC/T... |
| **10** | rs2395479 | 79318095 | 5063 | 2.291 | 1.644 | 3.194 | 9.908e-07 | 0.063 | T/C |
| **10** | rs2619642 | 79325583 | 5063 | 2.291 | 1.644 | 3.194 | 9.908e-07 | 0.063 | A/G |
| **10** | rs2670147 | 79321650 | 5063 | 2.291 | 1.644 | 3.194 | 9.908e-07 | 0.063 | C/G |
| **10** | rs2670164 | 79327231 | 5063 | 2.291 | 1.644 | 3.194 | 9.908e-07 | 0.063 | A/G |
| **10** | rs2673475 | 79322188 | 5063 | 2.291 | 1.644 | 3.194 | 9.908e-07 | 0.063 | C/T |
| **10** | rs3851041 | 79323531 | 5063 | 2.291 | 1.644 | 3.194 | 9.908e-07 | 0.063 | G/A |
| **10** | rs816863 | 79315276 | 5063 | 2.291 | 1.644 | 3.194 | 9.908e-07 | 0.063 | C/T |
| **10** | rs846101 | 79310532 | 5063 | 2.291 | 1.644 | 3.194 | 9.908e-07 | 0.063 | A/C |
| **10** | rs2673401 | 79325814 | 5062 | 2.291 | 1.644 | 3.193 | 9.976e-07 | 0.063 | T/C |
| **10** | rs816830 | 79264359 | 5059 | 2.274 | 1.632 | 3.167 | 1.182e-06 | 0.063 | T/A |
| **10** | rs816831 | 79264405 | 5059 | 2.274 | 1.632 | 3.167 | 1.182e-06 | 0.063 | C/T |
| **10** | rs816833 | 79267366 | 5061 | 2.279 | 1.634 | 3.179 | 1.225e-06 | 0.063 | T/C |
| **10** | rs816869 | 79308422 | 5063 | 2.268 | 1.628 | 3.16 | 1.297e-06 | 0.063 | A/G |
| **10** | rs35438549 | 79302463 | 5060 | 2.267 | 1.627 | 3.158 | 1.322e-06 | 0.063 | CAA/C... |
| **10** | rs816827 | 79291349 | 5060 | 2.267 | 1.627 | 3.158 | 1.322e-06 | 0.063 | A/G |
| **10** | rs816854 | 79302346 | 5060 | 2.267 | 1.627 | 3.158 | 1.322e-06 | 0.063 | A/T |
| **10** | rs816855 | 79297478 | 5060 | 2.267 | 1.627 | 3.158 | 1.322e-06 | 0.063 | C/T |
| **7** | rs79445594 | 12089652 | 5035 | 3.113 | 1.962 | 4.938 | 1.412e-06 | 0.024 | C/T |
| **11** | rs181415750 | 66979503 | 5044 | 3.788 | 2.203 | 6.512 | 1.46e-06 | 0.013 | G/A |
| **10** | rs1659784 | 79286614 | 5062 | 2.259 | 1.621 | 3.147 | 1.468e-06 | 0.063 | T/C |
| **18** | rs193153064 | 70085362 | 4982 | 3.842 | 2.221 | 6.645 | 1.47e-06 | 0.013 | T/C |
| **5** | rs114514212 | 59399245 | 5028 | 2.634 | 1.776 | 3.908 | 1.47e-06 | 0.035 | A/G |
| **10** | rs11306155 | 79254033 | 5063 | 2.256 | 1.62 | 3.144 | 1.517e-06 | 0.063 | C/C... |
| **10** | rs1248563 | 79248038 | 5063 | 2.256 | 1.62 | 3.144 | 1.517e-06 | 0.063 | T/C |
| **10** | rs1248566 | 79280527 | 5063 | 2.256 | 1.62 | 3.144 | 1.517e-06 | 0.063 | G/A |
| **10** | rs1711290 | 79253630 | 5063 | 2.256 | 1.62 | 3.144 | 1.517e-06 | 0.063 | T/G |
| **10** | rs703266 | 79277424 | 5063 | 2.256 | 1.62 | 3.144 | 1.517e-06 | 0.063 | A/G |
| **10** | rs816829 | 79263445 | 5063 | 2.256 | 1.62 | 3.144 | 1.517e-06 | 0.063 | T/G |
| **10** | rs816834 | 79268125 | 5063 | 2.256 | 1.62 | 3.144 | 1.517e-06 | 0.063 | C/T |
| **10** | rs816838 | 79274098 | 5063 | 2.256 | 1.62 | 3.144 | 1.517e-06 | 0.063 | C/T |
| **10** | rs816841 | 79275329 | 5063 | 2.256 | 1.62 | 3.144 | 1.517e-06 | 0.063 | A/C |
| **10** | rs816842 | 79276133 | 5063 | 2.256 | 1.62 | 3.144 | 1.517e-06 | 0.063 | A/G |
| **10** | rs816843 | 79276782 | 5063 | 2.256 | 1.62 | 3.144 | 1.517e-06 | 0.063 | A/G |
| **10** | rs816845 | 79278877 | 5063 | 2.256 | 1.62 | 3.144 | 1.517e-06 | 0.063 | T/C |
| **10** | rs816848 | 79251409 | 5063 | 2.256 | 1.62 | 3.144 | 1.517e-06 | 0.063 | G/A |
| **10** | rs816849 | 79251553 | 5063 | 2.256 | 1.62 | 3.144 | 1.517e-06 | 0.063 | C/T |
| **10** | rs816851 | 79253439 | 5063 | 2.256 | 1.62 | 3.144 | 1.517e-06 | 0.063 | A/G |
| **10** | rs816860 | 79260935 | 5063 | 2.256 | 1.62 | 3.144 | 1.517e-06 | 0.063 | T/C |
| **10** | rs843834 | 79268645 | 5063 | 2.256 | 1.62 | 3.144 | 1.517e-06 | 0.063 | A/G |
| **10** | rs865292 | 79262414 | 5063 | 2.256 | 1.62 | 3.144 | 1.517e-06 | 0.063 | C/A |
| **5** | rs147431808 | 59449854 | 5029 | 2.628 | 1.772 | 3.899 | 1.555e-06 | 0.035 | TA/T |
| **10** | rs1268510 | 79246052 | 5063 | 2.252 | 1.616 | 3.138 | 1.612e-06 | 0.063 | A/G |
| **1** | rs114930791 | 104367844 | 5040 | 3.734 | 2.178 | 6.401 | 1.656e-06 | 0.015 | T/C |
| **1** | rs182740455 | 104358369 | 5035 | 3.73 | 2.176 | 6.394 | 1.688e-06 | 0.015 | T/C |
| **5** | rs74859419 | 59328055 | 5026 | 2.614 | 1.762 | 3.878 | 1.813e-06 | 0.036 | G/A |
| **5** | rs78163262 | 59305008 | 5026 | 2.614 | 1.762 | 3.878 | 1.813e-06 | 0.036 | C/A |
| **11** | rs375601261 | 66981671 | 5046 | 3.731 | 2.171 | 6.41 | 1.876e-06 | 0.014 | A/G |
| **5** | rs140120203 | 59478552 | 5032 | 2.592 | 1.747 | 3.846 | 2.203e-06 | 0.036 | C/A |
| **6** | rs2350090 | 73577687 | 5040 | 3.818 | 2.191 | 6.651 | 2.247e-06 | 0.013 | A/C |
| **7** | rs76861468 | 12106392 | 5045 | 3.005 | 1.902 | 4.747 | 2.394e-06 | 0.024 | A/C |
| **3** | rs55636532 | 191912467 | 4995 | 3.495 | 2.077 | 5.883 | 2.469e-06 | 0.019 | C/T |
| **9** | rs10963602 | 18419150 | 4982 | 1.758 | 1.383 | 2.234 | 3.959e-06 | 0.204 | C/G |
| **7** | rs79921899 | 12187339 | 5031 | 3.013 | 1.883 | 4.823 | 4.299e-06 | 0.024 | T/A |
| **11** | rs145412681 | 67024683 | 5052 | 3.525 | 2.055 | 6.046 | 4.701e-06 | 0.014 | G/A |
| **9** | rs10963601 | 18419047 | 4974 | 1.743 | 1.374 | 2.212 | 4.821e-06 | 0.209 | C/T |
| **6** | rs138831395 | 92677130 | 5042 | 3.576 | 2.07 | 6.178 | 4.913e-06 | 0.014 | A/G |
| **10** | rs34880591 | 79307897 | 5059 | 2.129 | 1.539 | 2.945 | 4.995e-06 | 0.072 | GA/G |
| **7** | rs78655262 | 12412340 | 5054 | 2.899 | 1.835 | 4.578 | 5.029e-06 | 0.025 | G/T |
| **1** | rs187234497 | 165055013 | 5044 | 3.978 | 2.198 | 7.199 | 5.077e-06 | 0.012 | G/C |
| **7** | rs80059108 | 12312769 | 5048 | 2.891 | 1.83 | 4.565 | 5.304e-06 | 0.025 | A/G |

CHR = chromosome, BP = base pair (chromosome position), N = number of study participants, OR = odds ratio, L95 and U95 = lower and upper confidence interval, P = p-value, MAF = Minor allele frequency, GTPS = Genotypes minor/major allele, E = exponent of 10.

**Table S3.** The 60 variants within and flanking *ETV6, BDKRB2, MME, PRKCQ, SERPINE1,* and *F12* with the highest association to angioedema induced by angiotensin-converting enzyme inhibitor (ACEi) or angiotensin receptor blocker (ARB) treatment when cases were compared with all controls.

| CHR | SNP | BP | N | OR | L95 | U95 | P | GTPS | MAF  cases | MAF  controls | *Gene including 10000 BP flanking sequence* |
| --- | --- | --- | --- | --- | --- | --- | --- | --- | --- | --- | --- |
| 12 | rs113768037 | 12006062 | 5055 | 1.793 | 1.25 | 2.572 | 0.00153 | C/A | 0.108 | 0.065 | *ETV6* |
| 12 | rs73066136 | 12005502 | 5055 | 1.79 | 1.248 | 2.569 | 0.00157 | A/T | 0.108 | 0.065 | *ETV6* |
| 12 | rs10845413 | 12012106 | 5057 | 1.756 | 1.22 | 2.528 | 0.00247 | T/C | 0.105 | 0.065 | *ETV6* |
| 12 | rs73066167 | 12017753 | 5052 | 1.753 | 1.218 | 2.524 | 0.00252 | G/A | 0.104 | 0.064 | *ETV6* |
| 12 | rs73066169 | 12018349 | 5051 | 1.753 | 1.218 | 2.523 | 0.00253 | C/T | 0.104 | 0.064 | *ETV6* |
| 12 | rs73066171 | 12019081 | 5049 | 1.752 | 1.217 | 2.522 | 0.00255 | A/G | 0.104 | 0.064 | *ETV6* |
| 12 | rs113332016 | 12022013 | 5054 | 1.751 | 1.217 | 2.521 | 0.00256 | T/A | 0.104 | 0.064 | *ETV6* |
| 12 | rs73066159 | 12014774 | 5062 | 1.744 | 1.212 | 2.511 | 0.00277 | C/T | 0.104 | 0.065 | *ETV6* |
| 12 | rs55717959 | 12014808 | 5062 | 1.744 | 1.212 | 2.511 | 0.00277 | C/T | 0.104 | 0.065 | *ETV6* |
| 12 | rs111286385 | 12008218 | 5062 | 1.744 | 1.211 | 2.512 | 0.00279 | T/C | 0.105 | 0.065 | *ETV6* |
| 14 | rs4905462 | 96680574 | 5046 | 1.455 | 1.123 | 1.885 | 0.00452 | A/G | 0.222 | 0.165 | *BDKRB2* |
| 12 | rs2238130 | 12016008 | 5063 | 1.54 | 1.141 | 2.079 | 0.00478 | G/A | 0.165 | 0.118 | *ETV6* |
| 14 | rs4900315 | 96680903 | 5045 | 1.384 | 1.102 | 1.739 | 0.00519 | C/T | 0.341 | 0.276 | *BDKRB2* |
| 12 | rs58815511 | 12034979 | 4997 | 1.578 | 1.14 | 2.184 | 0.00598 | C/G | 0.137 | 0.093 | *ETV6* |
| 14 | rs4900314 | 96679203 | 5061 | 1.431 | 1.105 | 1.854 | 0.00665 | C/T | 0.222 | 0.167 | *BDKRB2* |
| 3 | rs3773896 | 154824685 | 5047 | 1.432 | 1.102 | 1.859 | 0.00716 | C/A | 0.222 | 0.168 | *MME* |
| 14 | rs4900312 | 96678974 | 5063 | 1.424 | 1.095 | 1.851 | 0.00834 | A/G | 0.214 | 0.161 | *BDKRB2* |
| 12 | rs76767018 | 12031783 | 4964 | 1.664 | 1.127 | 2.458 | 0.01043 | A/G | 0.089 | 0.057 | *ETV6* |
| 3 | rs17383987 | 154821909 | 5038 | 1.423 | 1.085 | 1.867 | 0.0109 | T/C | 0.204 | 0.154 | *MME* |
| 3 | rs3773899 | 154821486 | 5036 | 1.423 | 1.084 | 1.866 | 0.01094 | T/C | 0.204 | 0.154 | *MME* |
| 14 | rs8016905 | 96675933 | 5063 | 1.346 | 1.07 | 1.694 | 0.01124 | A/G | 0.33 | 0.271 | *BDKRB2* |
| 10 | rs12784332 | 6471961 | 5063 | 0.7548 | 0.6046 | 0.9425 | 0.01302 | T/C | 0.387 | 0.452 | *PRKCQ* |
| 10 | rs1889001 | 6472247 | 5063 | 0.7556 | 0.6051 | 0.9434 | 0.01336 | A/C | 0.387 | 0.452 | *PRKCQ* |
| 10 | rs7100817 | 6474384 | 5059 | 0.7559 | 0.6054 | 0.9439 | 0.01351 | A/G | 0.387 | 0.452 | *PRKCQ* |
| 10 | rs10906559 | 6475947 | 5022 | 0.7553 | 0.6039 | 0.9447 | 0.01394 | T/C | 0.367 | 0.43 | *PRKCQ* |
| 10 | rs10906558 | 6475866 | 5054 | 0.757 | 0.6063 | 0.9453 | 0.01403 | A/G | 0.387 | 0.451 | *PRKCQ* |
| 14 | rs2887499 | 96682378 | 5053 | 1.324 | 1.057 | 1.658 | 0.0145 | A/G | 0.367 | 0.307 | *BDKRB2* |
| 10 | rs10906557 | 6475698 | 5056 | 0.7589 | 0.6079 | 0.9475 | 0.01483 | C/A | 0.39 | 0.453 | *PRKCQ* |
| 14 | rs4905466 | 96684770 | 5057 | 1.446 | 1.073 | 1.949 | 0.01532 | C/T | 0.15 | 0.11 | *BDKRB2* |
| 10 | rs7070193 | 6477903 | 5052 | 0.7617 | 0.6095 | 0.9518 | 0.01665 | T/C | 0.387 | 0.449 | *PRKCQ* |
| 10 | rs35372351 | 6478770 | 5051 | 0.7617 | 0.6096 | 0.9519 | 0.01668 | A/AC | 0.387 | 0.449 | *PRKCQ* |
| 10 | rs4748073 | 6476945 | 5052 | 0.7619 | 0.6097 | 0.9521 | 0.01679 | T/C | 0.387 | 0.449 | *PRKCQ* |
| 3 | rs3773898 | 154824601 | 5052 | 1.392 | 1.061 | 1.826 | 0.01701 | G/C | 0.202 | 0.156 | *MME* |
| 12 | rs144602173 | 12027399 | 4972 | 1.641 | 1.091 | 2.466 | 0.01727 | T/C | 0.083 | 0.054 | *ETV6* |
| 3 | rs9827313 | 154825213 | 5063 | 1.387 | 1.058 | 1.819 | 0.01797 | A/G | 0.202 | 0.156 | *MME* |
| 12 | rs34232909 | 12033441 | 5034 | 1.625 | 1.084 | 2.435 | 0.01862 | A/AT | 0.081 | 0.053 | *ETV6* |
| 14 | rs4905465 | 96684515 | 5058 | 1.43 | 1.061 | 1.928 | 0.019 | C/G | 0.15 | 0.111 | *BDKRB2* |
| 3 | rs17449556 | 154820888 | 5009 | 1.402 | 1.057 | 1.861 | 0.01916 | A/G | 0.189 | 0.145 | *MME* |
| 12 | rs73066181 | 12023843 | 5008 | 1.615 | 1.081 | 2.415 | 0.01941 | A/C | 0.086 | 0.057 | *ETV6* |
| 12 | rs73054170 | 12028142 | 4962 | 1.636 | 1.082 | 2.475 | 0.01956 | C/A | 0.081 | 0.052 | *ETV6* |
| 12 | rs11832110 | 12033536 | 5037 | 1.655 | 1.083 | 2.529 | 0.01979 | T/C | 0.073 | 0.046 | *ETV6* |
| 14 | rs1959051 | 96676786 | 5047 | 1.429 | 1.058 | 1.929 | 0.01998 | A/G | 0.148 | 0.11 | *BDKRB2* |
| 14 | rs11627108 | 96678093 | 5049 | 1.427 | 1.056 | 1.927 | 0.02048 | A/G | 0.148 | 0.11 | *BDKRB2* |
| 10 | rs139183311 | 6460861 | 5044 | 0.7718 | 0.6182 | 0.9636 | 0.02216 | T/TACTC | 0.395 | 0.455 | *PRKCQ* |
| 10 | rs199777848 | 6460868 | 5036 | 0.7748 | 0.6205 | 0.9675 | 0.02435 | T/C | 0.395 | 0.454 | *PRKCQ* |
| 14 | rs4900313 | 96679050 | 5063 | 1.41 | 1.045 | 1.901 | 0.02443 | A/G | 0.15 | 0.113 | *BDKRB2* |
| 10 | rs582052 | 6469155 | 5063 | 0.7774 | 0.6234 | 0.9694 | 0.02534 | G/T | 0.396 | 0.454 | *PRKCQ* |
| 10 | rs4750438 | 6468622 | 5059 | 0.7776 | 0.6236 | 0.9696 | 0.02549 | C/T | 0.396 | 0.454 | *PRKCQ* |
| 10 | rs113399585 | 6499832 | 4985 | 2.108 | 1.096 | 4.057 | 0.02554 | C/T | 0.029 | 0.015 | *PRKCQ* |
| 10 | rs12413321 | 6467975 | 5061 | 0.7777 | 0.6237 | 0.9697 | 0.02554 | G/T | 0.396 | 0.454 | *PRKCQ* |
| 10 | rs10906534 | 6466845 | 5060 | 0.7777 | 0.6237 | 0.9698 | 0.02557 | A/C | 0.396 | 0.454 | *PRKCQ* |
| 3 | rs4467412 | 154804700 | 5025 | 1.387 | 1.04 | 1.849 | 0.02575 | T/C | 0.179 | 0.139 | *MME* |
| 3 | rs17443031 | 154813986 | 5063 | 1.28 | 1.03 | 1.59 | 0.02591 | T/C | 0.483 | 0.429 | *MME* |
| 3 | rs1836915 | 154809102 | 5063 | 1.367 | 1.028 | 1.818 | 0.03164 | C/T | 0.182 | 0.143 | *MME* |
| 12 | rs73286689 | 11822420 | 5058 | 2.242 | 1.072 | 4.69 | 0.03205 | T/C | 0.026 | 0.011 | *ETV6* |
| 14 | rs11847625 | 96686296 | 4974 | 0.7745 | 0.613 | 0.9785 | 0.03217 | C/G | 0.325 | 0.383 | *BDKRB2* |
| 12 | rs138361434 | 11910950 | 5043 | 1.825 | 1.052 | 3.166 | 0.03237 | G/GC | 0.041 | 0.023 | *ETV6* |
| 14 | rs11851152 | 96682227 | 5054 | 0.7776 | 0.6176 | 0.9792 | 0.03244 | C/G | 0.338 | 0.397 | *BDKRB2* |
| 3 | rs3773901 | 154812555 | 5059 | 1.265 | 1.018 | 1.574 | 0.03425 | A/T | 0.48 | 0.429 | *MME* |
| 3 | rs3773902 | 154812531 | 5059 | 1.265 | 1.018 | 1.574 | 0.03425 | C/T | 0.48 | 0.429 | *MME* |

CHR = chromosome, BP = base pair (chromosome position), N = number of study participants, OR = odds ratio, L95 and U95 = lower and upper confidence interval, P = p-value, MAF = Minor allele frequency, GTPS = Genotypes minor/major allele, E = exponent of 10.

**Table S4a.** Chromatin state model based on imputed data from the Roadmap Epigenomics project.^12^

| TssA | Active TSS | Promoter |
| --- | --- | --- |
| PromU | Promoter Upstream TSS |  |
| PromD1 | Promoter Downstream TSS 1 |  |
| PromD2 | Promoter Downstream TSS 2 |  |
| Tx5 | Transcribed - 5' preferential | Transcribed |
| Tx | Strong transcription |  |
| Tx3 | Transcribed - 3' preferential |  |
| TxWk | Weak transcription |  |
| TxReg | Transcribed & regulatory (Prom/Enh) |  |
| TxEnh5 | Transcribed 5' preferential and Enh |  |
| TxEnh3 | Transcribed 3' preferential and Enh |  |
| TxEnhW | Transcribed and Weak Enhancer |  |
| EnhA1 | Active Enhancer 1 | Enhancer |
| EnhA2 | Active Enhancer 2 |  |
| EnhAF | Active Enhancer Flank |  |
| EnhW1 | Weak Enhancer 1 |  |
| EnhW2 | Weak Enhancer 2 |  |
| EnhAc | Primary H3K27ac possible Enhancer |  |
| DNase | Primary DNase | Dnase |
| ReprPC | Repressed Polycomb | Repressed |
| Het | Heterochromatin |  |
| ZNF/Rpts | ZNF genes & repeats | Other |
| PromP | Poised Promoter |  |
| PromBiv | Bivalent Promoter |  |
| Quies | Quiescent/Low | Low |

**Table S4b.** Functional annotation analysis of 15 single nucleotide polymorphisms (SNPs) in high linkage disequilibrium (r^2^> 0**.**8) with the top *KCNMA1* SNPs in selected tissues relevant for angioedema. Annotations in the relevant epigenomes and the chromatin state model based on imputed data (25 state, 12 marks, 127 epigenomes) were obtained from the Roadmap Epigenomics Project.^12^

| **SNP** | **Skin** | | | **Mucosa** | | | | | **Eso-phagus** | **Aorta** | **Smooth muscle** | | | | **Endo-thelium** | **Transcription factor binding motifs altered** |
| --- | --- | --- | --- | --- | --- | --- | --- | --- | --- | --- | --- | --- | --- | --- | --- | --- |
| **rs816848** |  |  |  |  |  |  |  |  |  |  |  |  |  |  |  | GR,PU.1,SMC3 |
| **rs816849** |  |  |  |  |  |  |  |  |  |  |  |  |  |  |  | SPZ1 |
| **rs1711290** |  |  |  |  |  |  |  |  |  |  |  |  |  |  |  | AHR/AHR,LBP-1,MYC/MYC,RAD21 |
| **rs816860** |  |  |  |  |  |  |  |  |  |  |  |  |  |  |  |  |
| **rs816830** |  |  |  |  |  |  |  |  |  |  |  |  |  |  |  | DMRT7,DBX1,FAC1,FOXA,FOXD3,FOXP1,HNF1,HOXD10,MEF2,SOX,ZFP105 |
| **rs816831** |  |  |  |  |  |  |  |  |  |  |  |  |  |  |  |  |
| **rs816833** |  |  |  |  |  |  |  |  |  |  |  |  |  |  |  | MSX-1,NANOG,PRDM1 |
| **rs816827** |  |  |  |  |  |  |  |  |  |  |  |  |  |  |  | AP1,AP2,BAF155,BCL,Bach1,Bach2,GATA,GR,HMGN3,HMX,IRF,MAF/MAF,MEF2,MYC/MYC,NF-E2, NRF2,PRDM1,PAX2/PAX2,RXRA,STAT,TCF4,P300 |
| **rs1464111** |  |  |  |  |  |  |  |  |  |  |  |  |  |  |  | ETS/ETS,GR,MYF,TATA |
| **rs2719998** |  |  |  |  |  |  |  |  |  |  |  |  |  |  |  | ETS/ETS,GR,HNF4,MIZF,NFKB |
| **rs2253545** |  |  |  |  |  |  |  |  |  |  |  |  |  |  |  |  |
| **rs2253530** |  |  |  |  |  |  |  |  |  |  |  |  |  |  |  | DMRT4,PITX2 |
| **rs816838** |  |  |  |  |  |  |  |  |  |  |  |  |  |  |  | STAT,SMAD3 |
| **rs816863** |  |  |  |  |  |  |  |  |  |  |  |  |  |  |  | PAX-6 |
| **rs2928137** |  |  |  |  |  |  |  |  |  |  |  |  |  |  |  | HOXA5,SP1 |
| **Epigenome ID** | E055 | E056 | E126 | E075 | E077 | E101 | E102 | E110 | E079 | E065 | E076 | E078 | E103 | E111 | E122 |  |
| **Cell line** | Foreskin Primary Cell Fibro-blast skin01 | Foreskin Primary Cell Fibro-blast skin02 | Adult Dermal Fibro-blast Primary Cells | Colonic Mucosa | Duode-num Mucosa | Rectal Mucosa Donor 29 | Rectal Mucosa Donor 31 | Stomach Mucosa | Esopha-gus | Aorta | Colon Smooth Muscle | Duode-num Smooth Muscle | Rectal Smooth Muscle | Stomach Smooth Muscle | Umbilical Vein Endo-thelial Cells |  |

**
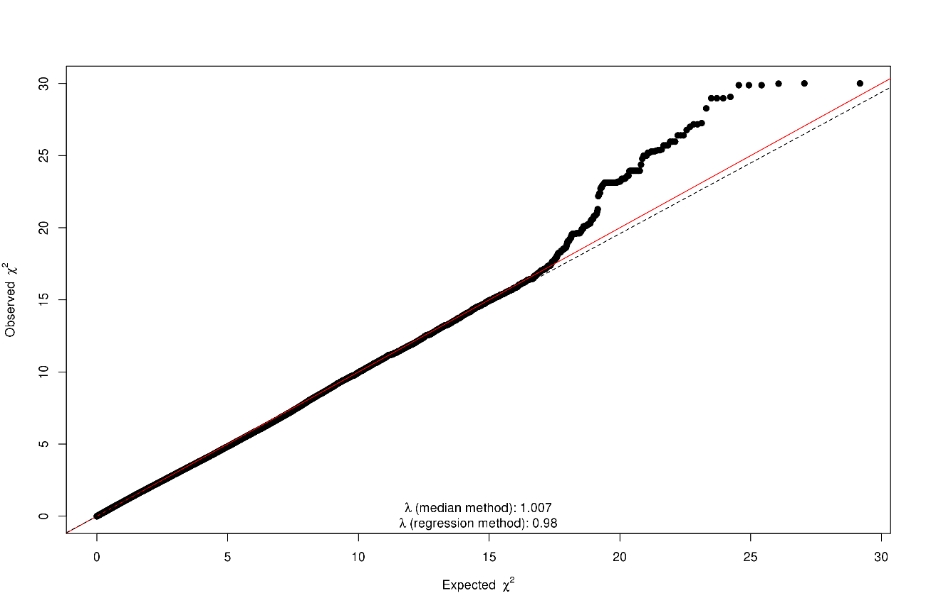
**

**Figure S1. Q-Q plot.** Analysis of all cases and controls in the discovery cohort after imputation, and adjusted by sex and genetic principal components 1-4. The quantile-quantile (Q-Q) plot characterizes the extent to which the observed distribution of Chi square values (black curve) follows the expected null distribution (x=y, dotted black line). The trend line (lambda=1.007, red) overlaps with the null distribution, except for a slight deviation in the right tail, which shows that the results are reliable.


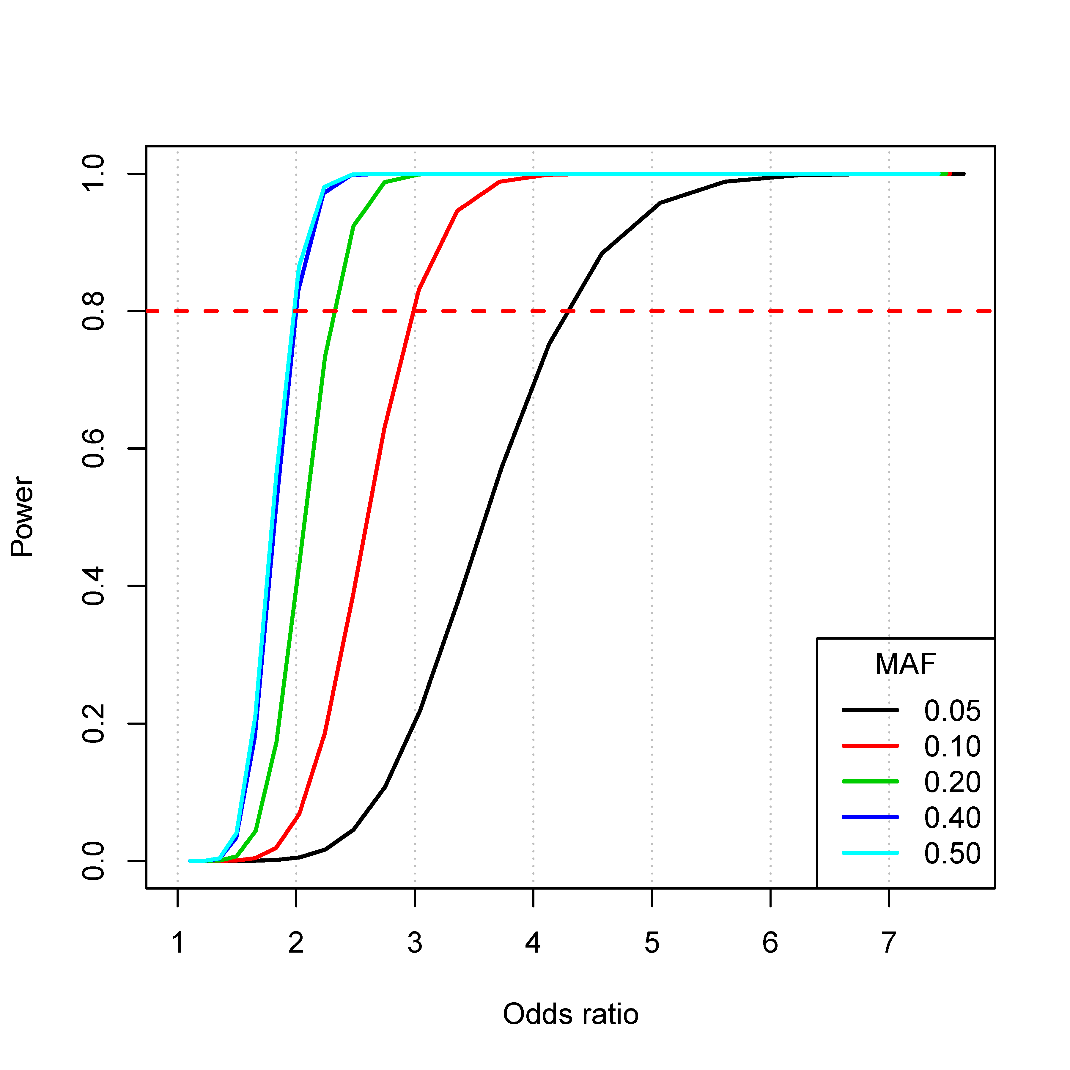


**Figure S2a. Power calculation** for 173 cases and 4890 controls, a genome-wide significance level of 5x10^-8^, an adverse drug reaction prevalence of 1% and an additive genetic model. For example, with a minor allele frequency (MAF) of 10%, we have 80% power to detect an odds ratio (OR) of 3.0.

**
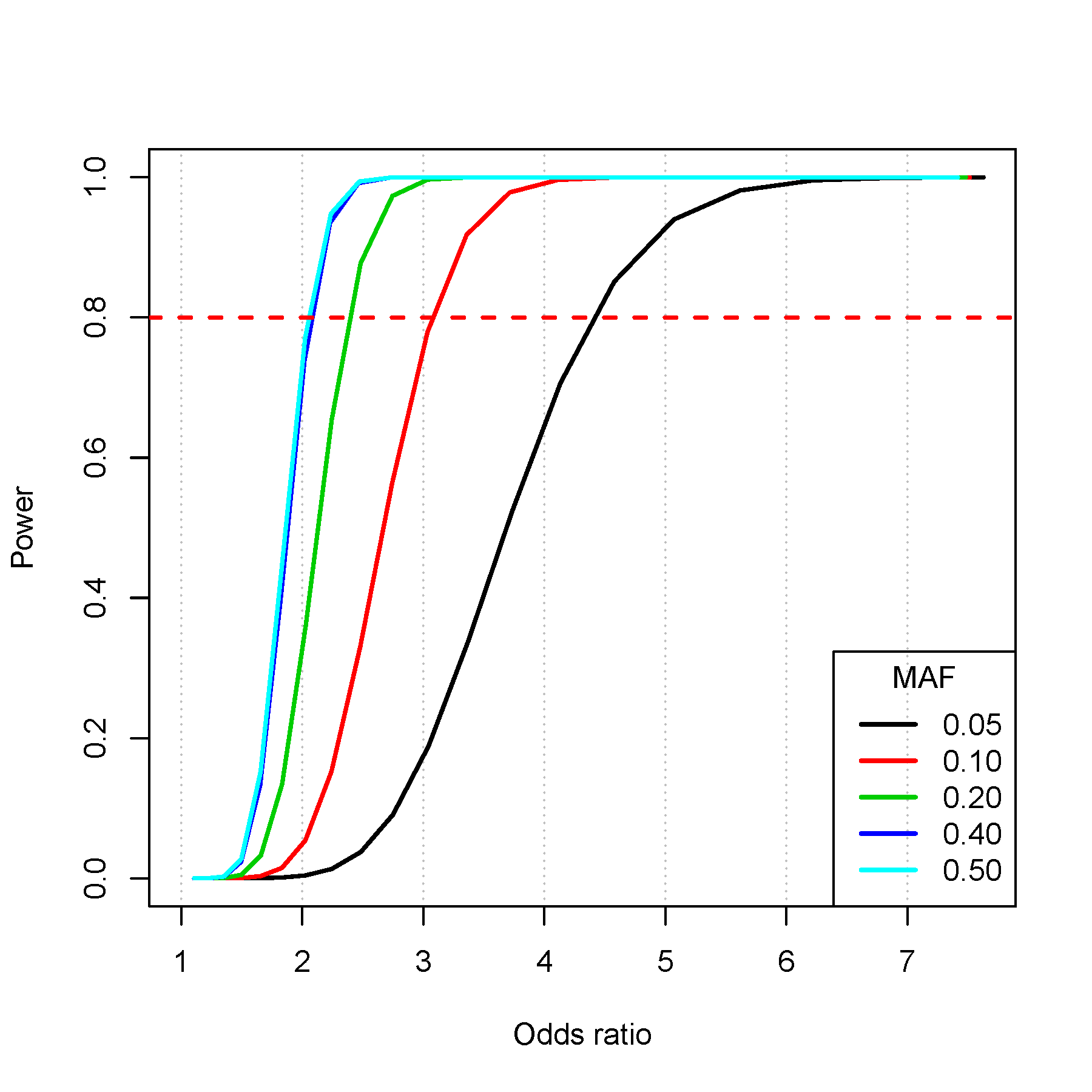
**

**Figure S2b. Power calculation** for 173 cases and 1345 controls, a genome-wide significance level of 5x10^-8^, an adverse drug reaction prevalence of 1% and an additive genetic model. For example, with a minor allele frequency (MAF) of 10%, we have 80% power to detect an odds ratio (OR) of 3.1.

**
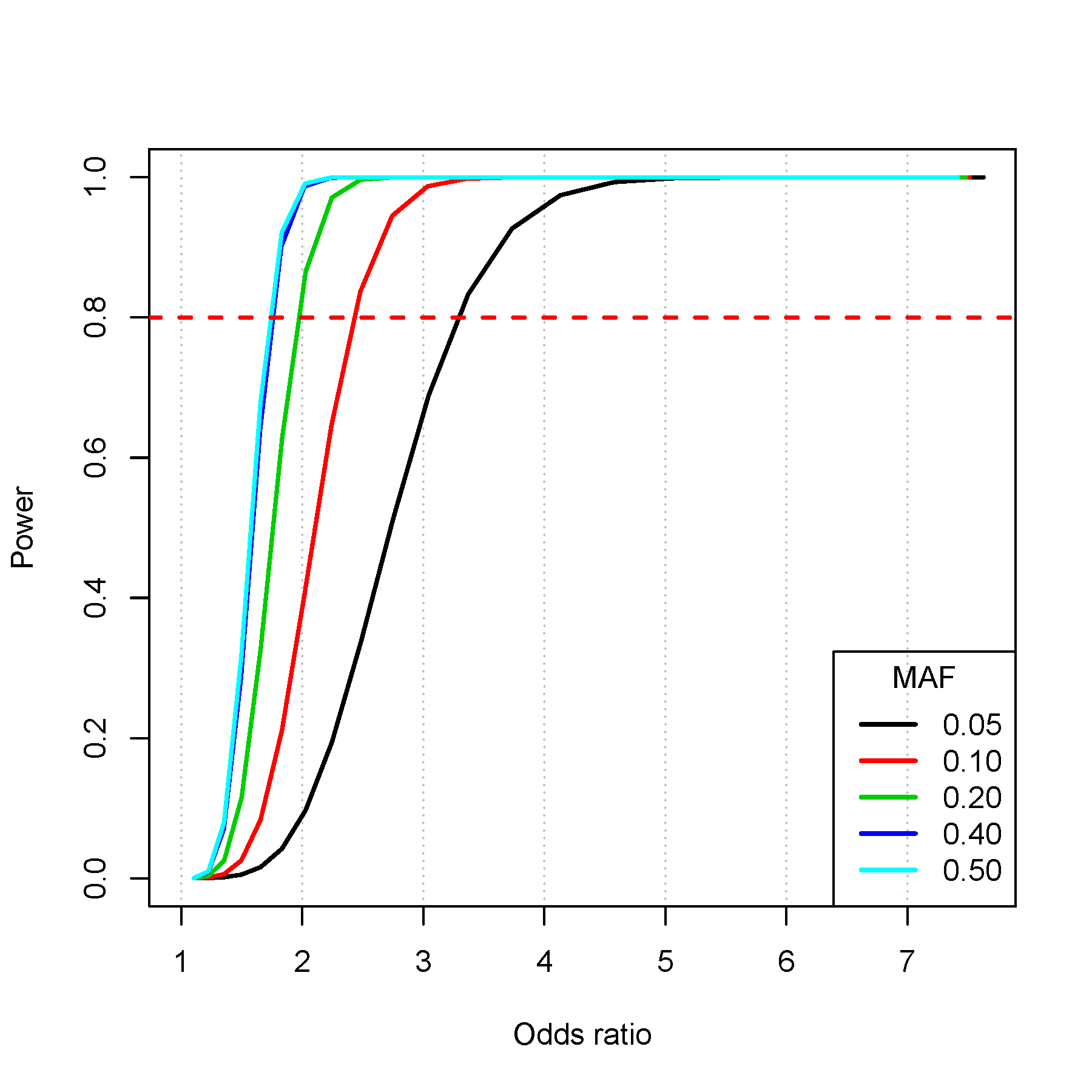
**

**Figure S3a. Power calculation** for 173 cases and 4890 controls, a candidate gene significance level of 2.89 X 10^-5^, an adverse drug reaction prevalence of 1% and an additive genetic model. For example, with a minor allele frequency (MAF) of 10%, we have 80% power to detect an odds ratio (OR) of 2.4.

**
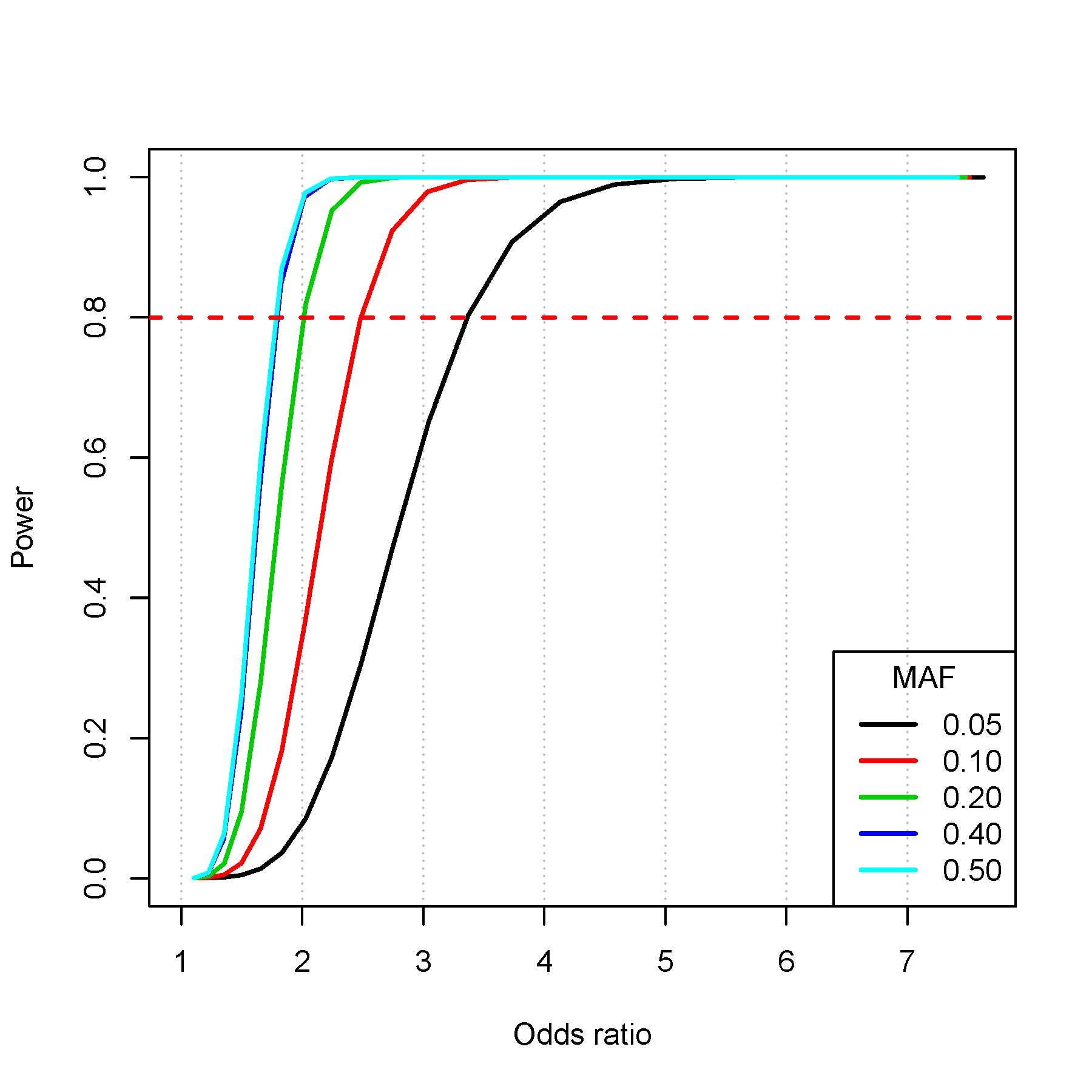
**

**Figure S3b. Power calculation** for 173 cases and 1345 controls, a candidate gene significance level of 2.89 X 10^-5^, an adverse drug reaction prevalence of 1% and an additive genetic model. For example, with a minor allele frequency (MAF) of 10%, we have 80% power to detect an odds ratio (OR) of 2.5.

**
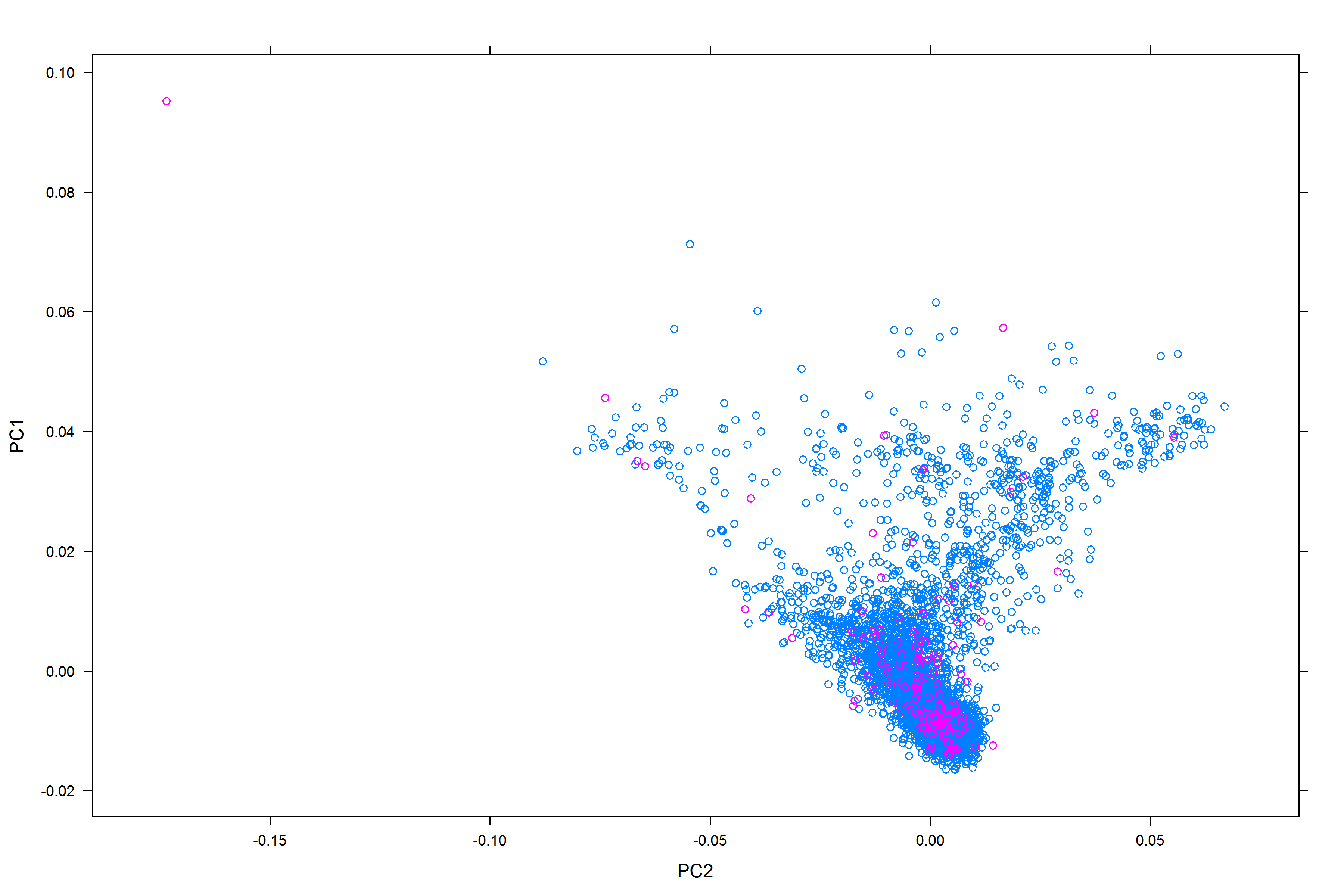
**

**Figure S4a. Principal component analysis (PCA).** Plot of the first two genetic principal components showing the population structure of the discovery cohort. Swedegene cases are in pink and TwinGene controls in blue.

**
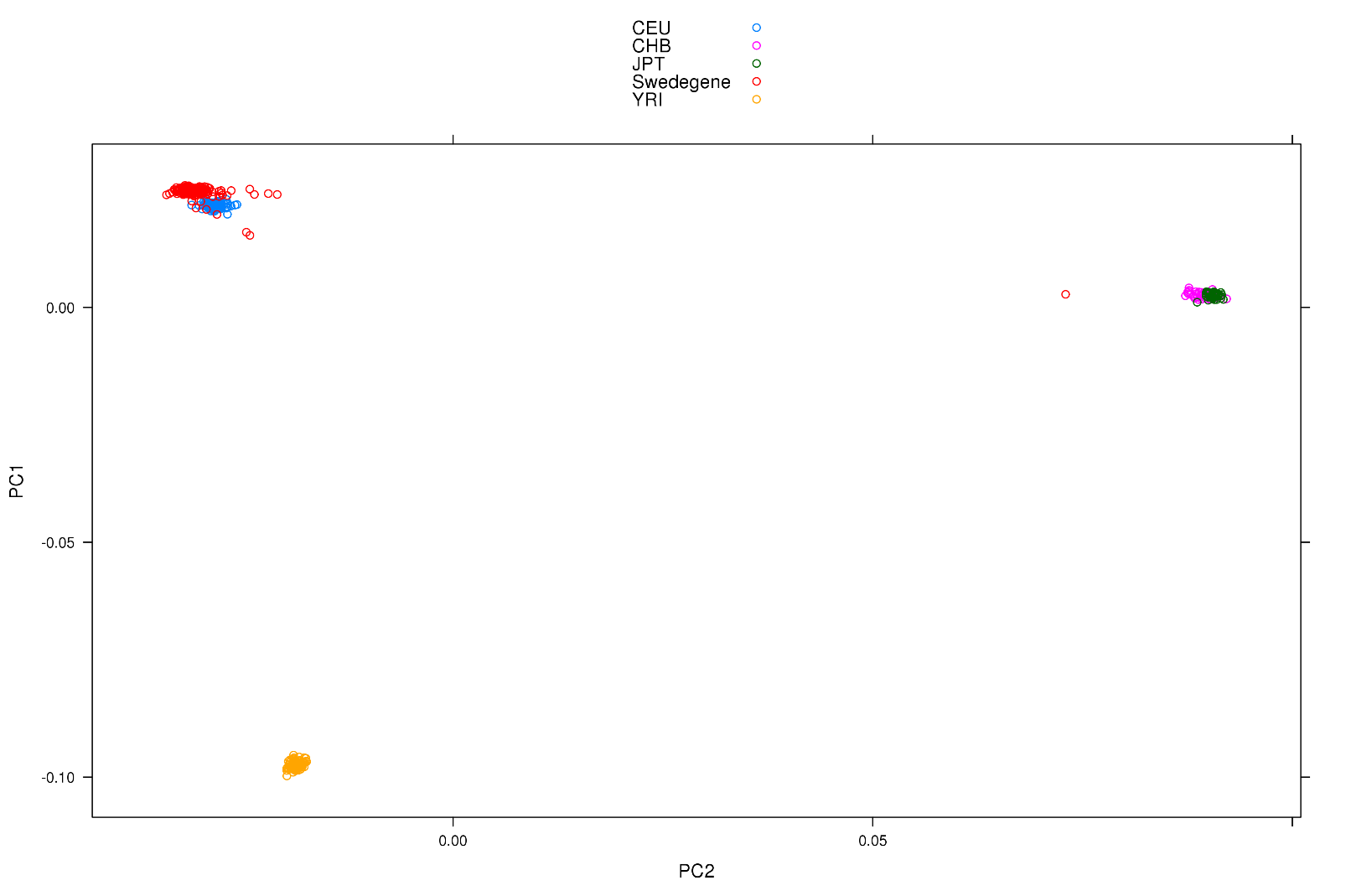
**

**Figure S4b. Principal component analysis (PCA) compared with HapMap data.** Population structure with the discovery cohort in red projected on HapMap coordinates using the first two genetic principal components. The HapMap populations are Utah residents with ancestry from Northern and Western Europe (CEU) in blue, Han Chinese in Beijing, China (CHB) in pink, Japanese in Tokyo, Japan (JPT) in green, and Yoruba in Ibadan, Nigeria (YRI) in yellow.^13^

**
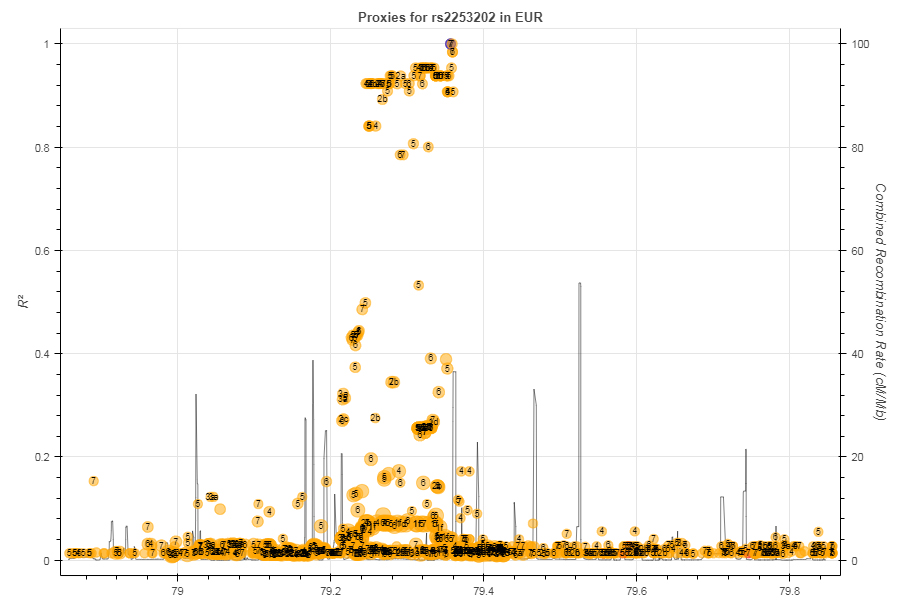
**

**Figure S5. Linkage disequilibrium plot in Europeans (EUR).** Plot of rs2253201 and all bi-allelic dbSNP variants in a window of plus or minus 500 kilobases (Kb). The X axis is the chromosomal coordinates and the Y axis is the pairwise R^2^ value with rs2253201 as well as the combined recombination rate from HapMap. Each point represents a proxy variant and the numbers represent the RegulomeDB scores. Plot generated with LDlink.^14^

**
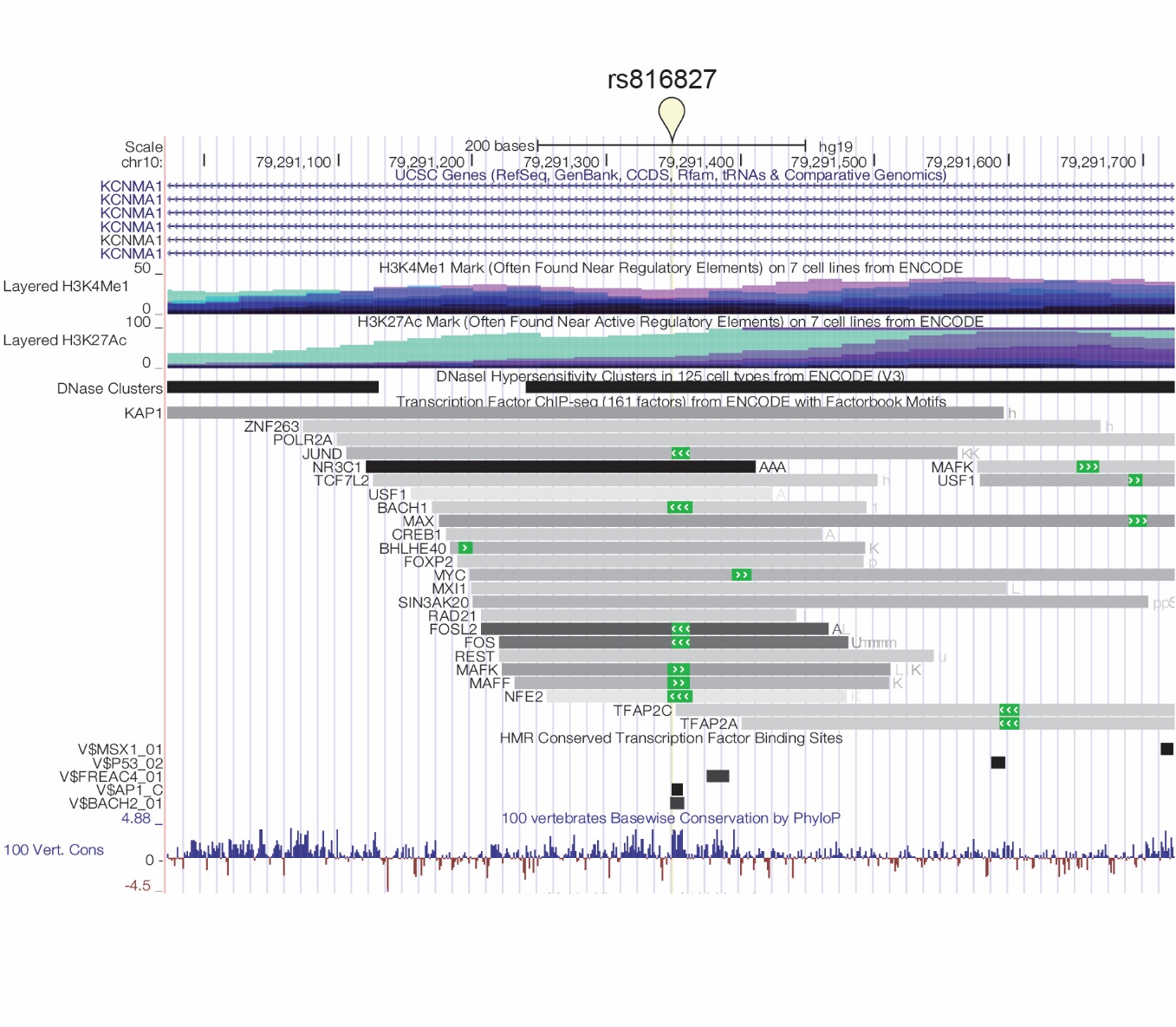
**

**Figure S6. Genome browser**. UCSC genome browser view for the genomic and transcription factor background of the *KCNMA1* variant rs816827.

**References**

1. Pare G, Kubo M, Byrd JB, et al. Genetic variants associated with angiotensin-converting enzyme inhibitor-associated angioedema. Pharmacogenet Genomics 2013;23:470-8.

2. Wadelius M, Marshall SE, Islander G, et al. Phenotype standardization of angioedema in the head and neck region caused by agents acting on the angiotensin system. Clin Pharmacol Ther 2014;96:477-81.

3. ATC/DDD Index 2019. 2019, at [www.whocc.no/atc_ddd_index/](file:///\\uuc-nas110.user.uu.se\medv-users$\miawadel\My%20documents\2019%20Mia%20HD\Swedegene%2019\Angioedema\Angioedema%20GWAS\Resubmission%20to%20TPJ\www.whocc.no\atc_ddd_index\).)

4. Magnusson PK, Almqvist C, Rahman I, et al. The Swedish Twin Registry: establishment of a biobank and other recent developments. Twin Res Hum Genet 2013;16:317-29.

5. National_Board_of_Health_and_Welfare. Swedish_Prescribed_Drug_Register <http://www.socialstyrelsen.se/statistik/statistikdatabas/lakemedel>: National_Board_of_Health_and_Welfare; 2019.

6. National_Patient_Register. 2019, at <https://www.socialstyrelsen.se/statistik-och-data/register/alla-register/patientregistret/>.)

7. Ludvigsson JF, Andersson E, Ekbom A, et al. External review and validation of the Swedish national inpatient register. BMC Public Health 2011;11:450.

8. International Statistical Classification of Diseases and Related Health Problems 10th Revision. at <https://icd.who.int/browse10/2016/en>.)

9. Poulsen FR, Munthe S, Soe M, Halle B. Perindopril and residual chronic subdural hematoma volumes six weeks after burr hole surgery: a randomized trial. Clin Neurol Neurosurg 2014;123:4-8.

10. Peters BJ, Rodin AS, Klungel OH, et al. Pharmacogenetic interactions between ABCB1 and SLCO1B1 tagging SNPs and the effectiveness of statins in the prevention of myocardial infarction. Pharmacogenomics 2010;11:1065-76.

11. Dujic T, Zhou K, Tavendale R, Palmer CN, Pearson ER. Effect of Serotonin Transporter 5-HTTLPR Polymorphism on Gastrointestinal Intolerance to Metformin: A GoDARTS Study. Diabetes Care 2016;39:1896-901.

12. Roadmap_Epigenomics_Consortium, Kundaje A, Meuleman W, et al. Integrative analysis of 111 reference human epigenomes. Nature 2015;518:317-30.

13. International_HapMap_Consortium, Altshuler DM, Gibbs RA, et al. Integrating common and rare genetic variation in diverse human populations. Nature 2010;467:52-8.

14. Machiela MJ, Chanock SJ. LDlink: a web-based application for exploring population-specific haplotype structure and linking correlated alleles of possible functional variants. Bioinformatics 2015;31:3555-7.
